# Functionally annotated electrophysiological neuromarkers of healthy ageing and memory function

**DOI:** 10.1002/hbm.26687

**Published:** 2024-04-23

**Authors:** Tibor Auer, Robin Goldthorpe, Robert Peach, Henry Hebron, Ines R. Violante

**Affiliations:** ^1^ School of Psychology University of Surrey Guildford UK; ^2^ Imperial College London London UK

**Keywords:** cognitive ageing, connectivity, electroencephalography, functionally annotated brain networks, memory, pipeline

## Abstract

The unprecedented increase in life expectancy presents a unique opportunity and the necessity to explore both healthy and pathological aspects of ageing. Electroencephalography (EEG) has been widely used to identify neuromarkers of cognitive ageing due to its affordability and richness in information. However, despite the growing volume of data and methodological advancements, the abundance of contradictory and non‐reproducible findings has hindered clinical translation. To address these challenges, our study introduces a comprehensive workflow expanding on previous EEG studies and investigates various static and dynamic power and connectivity estimates as potential neuromarkers of cognitive ageing in a large dataset. We also assess the robustness of our findings by testing their susceptibility to band specification. Finally, we characterise our findings using functionally annotated brain networks to improve their interpretability and multi‐modal integration. Our analysis demonstrates the effect of methodological choices on findings and that dynamic rather than static neuromarkers are not only more sensitive but also more robust. Consequently, they emerge as strong candidates for cognitive ageing neuromarkers. Moreover, we were able to replicate the most established EEG findings in cognitive ageing, such as alpha oscillation slowing, increased beta power, reduced reactivity across multiple bands, and decreased delta connectivity. Additionally, when considering individual variations in the alpha band, we clarified that alpha power is characteristic of memory performance rather than ageing, highlighting its potential as a neuromarker for cognitive ageing. Finally, our approach using functionally annotated source reconstruction allowed us to provide insights into domain‐specific electrophysiological mechanisms underlying memory performance and ageing.

**Highlights:**

- We provide an open and reproducible pipeline with a comprehensive workflow to investigate static and dynamic EEG neuromarkers.
- Neuromarkers related to neural dynamics are sensitive and robust.
- Individualised alpha power characterises cognitive performance rather than ageing.
- Functional annotation allows cross‐modal interpretation of EEG findings.

## Graphical abstract

We analysed a large open resting‐state EEG dataset and found that dynamic rather than static neuromarkers better capture cognitive ageing. We clarified that individualised alpha power reflects memory performance rather than ageing. Finally, we used functionally annotated source reconstruction to reveal domain‐specific electrophysiological mechanisms underlying memory performance and ageing.

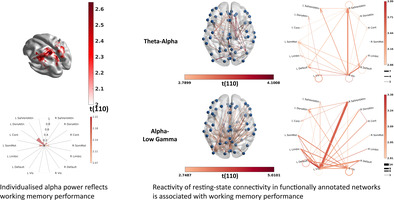

## 1 INTRODUCTION

A major accomplishment of the 20th century was the remarkable gain in global life expectancy, particularly in industrialised countries, which saw a rise of approximately 30 years (Christensen et al., 2009). However, age is also the strongest risk factor for many chronic diseases, including cancer and cardiovascular and neurodegenerative diseases (Niccoli & Partridge, 2012). Thus, understanding normal brain ageing and developing interventions that maintain cognitive function is paramount to support a better quality of life throughout one's lifespan. Among the earliest and extensively studied cognitive changes associated with ageing is the alteration of memory (Reid & MacLullich, 2006), which also occurs in individuals ageing typically without any evidence of dementia (James et al., 2008). Accordingly, as many as half of healthy older adults report worrying about their everyday memory (Jonker et al., 2000).

Planning successful intervention regimes requires understanding both non‐pathological and pathological changes associated with ageing and cognitive decline, alongside the identification of robust and reliable biomarkers (Belleville & Bherer, 2012; Gallen & D'Esposito, 2019; Simpraga et al., 2017). With the rise of open science and data sharing initiatives, there is now increased access to large datasets rich in phenotypic information (Collerton et al., 2007; Taylor et al., 2017), triggering several biomarker projects (Cole & Franke, 2017; Dickerson & Wolk, 2012; Engemann et al., 2020; Martin‐Ruiz et al., 2011). Among the potential biomarkers, functional brain imaging metrics have increasingly emerged as promising options to monitor and identify reliable markers of brain development, ageing and disease (Foo et al., 2021; Grady, 2012; Jack et al., 2017; Nyberg et al., 2010; Tooley et al., 2022). Functional MRI has been widely used in neuroimaging studies and provides valuable information. However, it has inherent limitations, particularly as an indirect measure of neural activity, with poorer temporal resolution that hinders the characterisation of the fast dynamic properties of neural processes. In contrast, electrophysiological techniques, such as magnetoencephalography (MEG) and electroencephalography (EEG) are better suited to investigate temporal and spectral properties of neural activity across the lifespan and in the context of diseases. Recent studies have highlighted their importance in understanding the neural changes underlying Alzheimer's Disease (AD) (Maestú et al., 2019) and their potential in neuromarker research (Engemann et al., 2020). Furthermore, EEG has the added advantage of being portable, easy to implement and an affordable imaging technique.

The high temporal resolution of M/EEG allows for the comprehensive characterisation of neural oscillations, providing insights into both normal and pathological brain function (Schnitzler & Gross, 2005). These oscillations are conventionally categorised into five classical frequency bands: delta (1–4 Hz), theta (4–7 Hz), alpha (8–13 Hz), beta (14–30 Hz), and gamma (>31 Hz). Alpha oscillations, in particular, constitute a robust electrophysiological characteristic of the awake human brain (Nunez & Srinivasan, 2009). Reduction in alpha power and alpha reactivity, defined as the reduction of alpha power upon opening the eyes, characterises not only non‐pathological ageing but also pathological cognitive decline (Babiloni et al., 2006; Babiloni et al., 2022). These findings have been substantiated in a recent meta‐analysis (Lejko et al., 2020) and have been associated with performance in memory, language, and executive functions (van der Hiele et al., 2008). Although some studies have reported evidence of age‐related changes in other frequency bands, these findings are less conclusive (Trammell et al., 2017). A common limitation in previous studies is the adoption of fixed boundaries between frequency bands, which can bias findings in populations with different characteristics, such as age (Cohen, 2021). Indeed, recent studies have highlighted the importance of considering individual alpha frequency when comparing power and brain connectivity differences between younger and older adults. They demonstrate that age‐related decreases in alpha frequency can bias findings in power and connectivity metrics against older adults (Jabès et al., 2021; Scally et al., 2018).

Functional connectivity measures, which commonly assess temporal correlations between time series data from two or more independent M/EEG channels or sources, have become increasingly recognised as key metrics for understanding brain regions' communication and activity coordination, as well as identifying potential neuromarkers of ageing and disease (Engels et al., 2015; Javaid et al., 2022; Schoonhoven et al., 2022). Consequently, numerous studies have reported age‐related changes in connectivity across various frequency bands, including increased connectivity in the beta band and decreased connectivity in other bands (Moezzi et al., 2019), increased connectivity in theta and beta bands, and age‐ and cognition‐related decreases in the alpha band (Chow et al., 2022). Additionally, cross‐frequency coupling has shown substantial predictive power in pathological cognitive decline (Musaeus et al., 2020). However, meta‐analyses revealed considerable methodological variation in functional connectivity studies, which causes significant level of inconsistency, and even contradictory findings (Lejko et al., 2020; Mahjoory et al., 2017). The vast methodological variations emphasise the importance of carefully constructed, reproducible, and shareable processing pipelines.

Furthermore, it is important to consider how we represent and summarise EEG findings to increase interpretability and facilitate comparison across different imaging modalities. In the fMRI literature, the widespread use of brain network parcellations based on structural and functional properties has contributed to the interpretability of activation patterns (Dewiputri et al., 2021). This is especially important in neuromarker research, as it strongly influences the mechanistic understanding and translational potential of the neuromarkers. While studies have demonstrated significant similarity between fMRI and M/EEG resting state networks (Brookes et al., 2011; Custo et al., 2017; Hillebrand et al., 2012), a network‐based approach for reporting and interpreting the functional relevance of electrophysiological findings is scarcely used (but see Choi et al., 2021; Zhang et al., 2021).

In this study, we leveraged the recently published Leipzig Study for Mind–Body‐Emotion Interactions (LEMON) dataset (Babayan et al., 2019), which includes, among other metrics, resting‐state EEG, and memory performance assessment in over 200 young and older adult participants. Our aim was to investigate electrophysiological neuromarkers of healthy ageing and their association with memory function. Resting‐state activity provides valuable insights into the intrinsic characteristics of the brain's neural architecture (Fox & Raichle, 2007), which can reflect individual differences in cognitive function (Zou et al., 2013) and discriminate pathological conditions (Zhang et al., 2021). Resting‐state data is particularly valuable in neuromarker research due to its ease of implementation, not requiring active participant engagement. This makes it more accessible and less burdensome in clinical settings, allowing for utilisation in longitudinal studies and identifying unique neurobiological signatures. Previous studies using the LEMON dataset have already begun demonstrating its utility in revealing age‐related changes in brain function. For example, studies have already shown a decrease in signal variability (SD) and power in the lower frequencies (1–12 Hz) in sources related to the default mode network (DMN), as well as an age‐related increase in signal variability and power in the higher frequencies (15–25 and 12–30 Hz, respectively) in sources in the central frontal and temporal regions (Kumral et al., 2020; Zhong et al., 2020).

Here, we expand on previous studies conducted on the LEMON dataset and other datasets by investigating several electrophysiological neuromarkers and their association with healthy ageing and memory performance within the same participants. We included both resting state conditions, i.e., eyes open (EO) and eyes closed (EC), using two different approaches. (a) By averaging their activity (mEOEC), we increased statistical power to identify features consistent across brain states, likely corresponding to individual traits (Engemann et al., 2022). (b) Calculating their ratio (EC/EO) as a marker of reactivity, however, can offer insights into changes in brain dynamics. This latter measure has been used in studies exploring cognitive decline (Barry & de Blasio, 2017) and predicting cognitive performance (van der Hiele et al., 2008). We investigated power and functional connectivity, both within‐ and across frequency bands, for both approaches. Given the growing recognition of individualised frequency bands, we considered how individual variations affected age‐ and memory‐related findings on activity and connectivity metrics. All analyses were performed at the source level, thus obtaining a more fine‐grained topographic distribution of the features, which is important in predicting cognitive ageing (Engemann et al., 2020). Finally, we interpret the results on the level of neuropsychologically meaningful networks to allow a more integrative view and a more direct link between the brain and the behaviour. To support open and reproducible science, we implemented our analyses using a configurable and scalable neuroimaging pipeline (Cusack et al., 2015).

## 2 METHODS

### 2.1 Participants

This study used data from the Leipzig Study for Mind–Body–Emotion Interactions (LEMON) project (Babayan et al., 2019), including 227 participants (82 female) divided into nine pre‐defined age groups with age ranges centred at 22.5, 27.5, 32.5, 37.5, 57.5, 62.5, 67.5, 72.5, and 77.5. Participants were further labelled as “young” (20–40 years old) and “older” (55–80 years old). Exclusion criteria included ongoing substance misuse, neurological disorders, malignant disease, cardiovascular disease, psychiatric illness requiring inpatient treatment, pregnancy, claustrophobia, metallic body implants including tattoos, tinnitus, hypertension, recent involvement in research or advanced psychology degrees, and certain medications including those acting on the central nervous system, chemotherapy, and psychopharmacological medicines. Data collection was in accordance with the Declaration of Helsinki and was approved by the medical faculty ethics committee at the University of Leipzig (reference 154/13‐ff ).

### 2.2 Behavioural data

Participants completed a cognitive test battery and the resting state (rs)‐EEG in separate sequential daily sessions. From the test battery, we selected the two tests that directly measured memory function, i.e., an adapted version of the California Verbal Learning Test (CVLT‐II; Niemann et al., 2008) and the 2‐Back task from the Test of Attentional Performance (TAP; Ziemmermann & Fimm, 1992; Ziemmermann & Fimm, 2002). The CVLT‐II was done first, followed by the TAP as a 20‐min filler task, and the CVLT‐II delayed recall condition after. TAP was employed as a filler task as it is unlikely to interfere with verbal learning (Babayan et al., 2019).

The CVLT‐II was used to assess participants' verbal learning and episodic memory. Participants were acoustically presented with 16 words and instructed to remember them. They were then presented with this same list and asked to immediately recall its contents five consecutive times. This was followed by several different recall conditions. In the interference condition, participants learned a new list once and immediately recalled the original list. In the cued condition, participants were presented with four categories and asked to recall which words from the original list correspond with which category. In the delayed condition, participants were asked to recall the original list after a delay of 20 min, both with and without category cues. In the recognition condition, participants were presented with a new list of words and asked to determine which of these were on the original list. Cued conditions were used to assess associative memory functioning, whilst performance across the other conditions assessed episodic memory.

The 2‐Back task from the TAP was used to assess working memory. This task presented visually to participants on a computer screen, serially showed a list of numbers (1–9) for 5 min. Participants were required to press a button if the number currently on the screen matched the one they saw two numbers prior. Accuracy, omissions, and reaction times were recorded for each participant to assess their performance.

Composite measures of associative (AM), episodic (EM), and working memory (WM) were created by extracting scores from the CVLT‐II and TAP. These composite scores were favoured over individual test scores to allow for a more comprehensive memory assessment of each domain. A composite episodic memory score was created by factor analysing CVLT‐II recall on the first and fifth trials, the sum of correct recalls from the first to fifth trial, recall after a short delay, delayed recall by 20 min, and recognition of learned words. All measures correlated above 0.4, Kaiser–Meyer–Olkin's measurement of sampling adequacy considerably exceeded 0.5 at 0.81, and Bartlett's test of Sphericity was significant (χ^2^(15) = 1273.51, *p* < .001), all of which indicated good factorizability. From this, one principal component with an Eigenvalue above 1 emerged, which accounted for 71.24% of the variance across episodic memory scores. A composite associative memory score was similarly created by factor analysing word recall after a short delay with category cues present and delayed recall by 20 min with category cues present. Measures were highly correlated, given the smaller number of variables, while the Kaiser–Meyer–Olkin coefficient was lower but still acceptable at 0.5 (Hadia et al., 2016), and Bartlett's test of Sphericity was significant (χ^2^(1) = 425.50, *p* < .001). From this analysis, one principal component with an Eigenvalue >1 emerged, which accounted for 96.16% of the variance across associative memory scores. An index score was created to assess working memory faculties measured with the accuracy on the 2‐Back TAP task. This index was calculated by subtracting the number of incorrect responses from the number of correct responses and dividing the total by 15 (the number of possible correct responses).

### 2.3 EEG

#### 2.3.1 Data acquisition

Sixteen min of rs‐EEG data were acquired for 216 participants using a BrainAmp MR plus amplifier with 62‐channel active ActiCAP electrodes (Brain Products GmbH, Germany). Electrodes were placed according to the 10–10 localisation system, referenced to the FCz electrode, and the ground electrode was placed on the sternum. Skin electrode impedance was kept below 5 KΩ. During EEG data acquisition, EEG amplitude resolution was set to 0.1 μV, data were digitised at a sampling rate of 2500 Hz, and recorded with an online band‐pass filter between 0.015 Hz and 1 kHz.

Rs‐EEG data were collected over 16 blocks, each lasting 60 s. Blocks were divided into eight eyes‐open (EO) and eight eyes‐closed (EC) conditions interleaved. Changes between blocks were announced using Presentation software (version 16.5, Neurobehavioral Systems Inc., Berkeley, CA). Participants were seated in front of a computer screen and asked to stay awake during EEG data acquisition. During the EO blocks, they were asked to fixate their gaze on a black cross presented on a white background.

#### 2.3.2 Pre‐processing

We opted for using the raw data instead of the available pre‐processed EEG data for two main reasons. The first was that the published pre‐processed data had been band‐passed with 1–45 Hz, thus removing information on high‐frequencies which may hold important information regarding cognition and age‐related changes in brain function (Başar, 2013; Herrmann et al., 2004; Herrmann et al., 2010). The second was that this allowed us to implement an open access processing workflow, which allows us better control over data quality, larger transparency, and flexibility in pre‐processing.

Raw rs‐EEG was pre‐processed using Automatic Analysis (*aa*, version 5.6; https://automaticanalysis.github.io; Cusack et al., 2015) running on MATLAB R2020a (Mathworks Inc., Natick, MA). The workflow (Figure S1) included pre‐processing using EEGLab (version 2020.0; Delorme & Makeig, 2004) and FieldTrip (git revision 666b4e3; Oostenveld et al., 2011). Raw rs‐EEG data were initially down‐sampled from 2500 to 250 Hz and high‐pass filtered with 1 Hz. Line noise was removed using a band‐pass filter at 50 and 100 Hz with a bandwidth of 10 Hz. Artefactual channels and data segments were removed using Artifact Subspace Reconstruction (Chang et al., 2018), and data were re‐referenced to a common average reference before further data processing. Further pre‐processing included Independent Component Analysis using the AMICA algorithm (Delorme et al., 2012; Palmer et al., 2007) followed by automated IC selection using IClabel (Pion‐Tonachini et al., 2019) and dipole fitting to inform the classification of components. Finally, data were divided into EO and EC conditions and epoched with a 2‐sec interval within each condition (maximum eight blocks × 30 = 240 epochs). Following pre‐processing, 163 participants' EEG data with at least 100 clean epochs were retained for further analysis to avoid large between‐subject variability in the reliability of the various power and connectivity estimates.

#### 2.3.3 Data analysis

Data analysis was conducted using FieldTrip (git revision 666b4e3; Oostenveld et al., 2011) as integrated into *aa* (Figure S1). Power spectral density (PSD) was calculated using fast Fourier transforms across the spectrum between 1 and 120 Hz after ‘tapering’ the data with the Hanning window. Across all participants, a standard, highly detailed Finite Element Method volume conduction model was used to solve the forward problem (Huang et al., 2016) using the SimBio toolbox as integrated into FieldTrip (Vorwerk et al., 2018). The source model was created based on the cortical sheet of each participant as constructed with FreeSurfer and downsampled to around 4000 tessels using Connectome Workbench (https://www.humanconnectome.org/software/connectome-workbench). Source activity was reconstructed by using exact low‐resolution brain electromagnetic tomography (eLORETA) as implemented in FieldTrip (Pascual‐Marqui, 2007). The leadfield matrix and the source filter were generated between the modelled cortical sources and the EEG channels and were used to compute the abovementioned time‐frequency decomposition at the source level.

The epoched signal was computed at the source level and averaged for regions of the Desikan‐Killiany‐Tourville (DKT) atlas (Desikan et al., 2006). The Freesurfer parcellation annotated 62 regions according to the DKT atlas; however, 11 of them (the entorhinal, the cingulate isthmus, the medial orbitofrontal, the parahippocampal, and the pars orbitalis of the inferior frontal bilaterally, as well as the right posterior cingulate) failed to be mapped to the source model thus leaving 51 virtual channels. Band‐limited power spectrums were calculated by averaging the PSD according to standard EEG frequency bands (delta: 1–3 Hz; theta: 4–7 Hz; alpha: 8–13 Hz; beta: 14–32 Hz; lower gamma: 33–80 Hz; upper gamma: 81–120 Hz) before statistical analysis. For connectivity, time‐frequency decomposition was averaged according to 33 bins with increasing width (delta: 6 bins; theta: 7 bins; alpha: 6 bins; beta: 5 bins; lower gamma: 5 bins; upper gamma: 4 bins) and two measures were computed between virtual channels. These were the within‐frequency connectivity, as characterised by the debiased weighted phase lag index (wPLI) (Vinck et al., 2011), and cross‐frequency connectivity as characterised by the phase locking value (PLV) (Schmidt et al., 2014). In a recent study, Siebenhühner and co‐workers demonstrated the reliability and biological plausibility of these measures (Siebenhühner et al., 2020), as well as their correspondence with cognitive performance.

In addition to considering standard EEG frequency bands, we estimated individual bands using a combination of extended Better OSCillation detection (eBOSC) (Kosciessa et al., 2020) and Fitting Oscillations & One Over F (FOOOF) (Donoghue et al., 2020). eBOSC uses a 6‐wave wavelet transform across the spectrum to calculate PSD for individual band detection. On the other hand, FOOOF operates by parameterising a PSD model by fitting Gaussian curves to capture band‐limited power spectra as peak‐like deviations from the background activity. After calculating the PSD across the spectrum between 2 and 80 Hz, a FOOOF model was fitted to detect up to six Gaussian curves with a peak width between 1 and 6 Hz and a minimum peak height of 0.05 a.u. or 1.5 SD, whichever is higher. The band estimates (peak frequency and bandwidth) were averaged across channels to calculate the individual bands. The procedure provides stable estimates for alpha and beta bands within the standard bands (see above). Theta band was shifted accordingly while keeping its original bandwidth; however, the delta band's width was adjusted to correspond to all frequencies below the theta band. Gamma bands were unaffected. Individual variations in band frequencies are accounted for in all results reported in the main text, while corresponding results without band individualisation are reported in the supplementary material for comparison.

#### 2.3.4 Combined neural measures

Averaging measures for EC and EO conditions (mean (*m*) ECEO, calculated as $\frac{\mathrm{EC}+\mathrm{EO}}{2}$) allows the investigation of neural features stable across the two states. The literature on defining EC/EO reactivity is not conclusive, and various approaches have been reported from simple difference (Bellato et al., 2020), through ratio (Fonseca et al., 2011), to normalised difference (Wan et al., 2019); usually without detailed justification. Considering a report of a linear relationship between EC and EO estimates in all frequency bands (Barry & de Blasio, 2017) also seen in our data (*not shown*), we decided to use their ratio (i.e., $\frac{\mathrm{EC}}{\mathrm{EO}}$) as a measure of EC/EO reactivity.

The combinations of neural measures have been performed at the last stage of the analysis before statistics. For example, source‐level power estimates have been calculated for the EC and the EO conditions separately, and then these source‐level estimates have been combined (average and ratio, respectively) to obtain the final features we entered in the statistical analysis.

#### 2.3.5 Statistical analysis

Behavioural data were analysed with R version 3.6.0 (26 April 2019). Since none of the behavioural data showed a normal distribution (AM: W = 0.94, *p* < .001; EM: W = 0.97, *p* = .003; WM: W = 0.81, *p* < .001), we used Mann–Whitney *U* tests and Kruskal–Wallis tests when comparing “young” and “older” groups and the nine pre‐defined age groups, respectively. Effect sizes have been interpreted according to Funder and Ozer (2019).

The effect of age and the neural correlate of the associative, episodic, and working memory domains were tested on all estimates, i.e., the time‐frequency decomposition and the within‐ and cross‐frequency connectivity. The effect of age was tested by means of linear regression using the interval variable of age groups as the independent variable. The neural correlates of memory domains were tested by means of linear regression using the composite measures as the independent variable. Due to the strong linear relationship between age and memory performances (see Results), these linear regressions have been conducted for the “young” and “older” groups separately. Also, the effect of age on memory performances has been accounted for by the orthogonalisation of memory performances with respect to age within each group.

Statistical inference was calculated by means of nonparametric Monte‐Carlo estimation of the significance probabilities as implemented in FieldTrip. For all estimates, statistical significance was calculated based on 1000 iterations of threshold‐free cluster enhancement (TFCE), and a significance threshold of *p* = .05 was employed.

#### 2.3.6 Graphs for cluster‐forming rules for functional connectivity

All inferencing in spatially resolved neuroimaging must apply some correction for multiple comparisons. While engineering advancement allows data with higher resolution, it also increases the number of tests to an extent when more traditional techniques, such as Bonferroni correction leads to a higher chance of false negatives. For connectivity, the increase of the number of tests is exponential with the increase in resolution, i.e., the number of channels, as 10 times more channels result in 10^2^ times more channel combinations for connectivity. TFCE‐ and other cluster‐based statistics are robust inferential procedures offering greater freedom and sensitivity (Maris & Oostenveld, 2007). They, however, require clear rules on cluster formation, i.e., which data points can form a cluster. If we consider time‐frequency decomposition, where the data are acquired from single channels, a cluster‐forming rule can be defined based on the topographic distribution of the channels based on the assumption that data from channels spatially closer to each other are likely to be more similar (Frömer et al., 2018). However, defining a cluster‐forming rule for channel combinations for connectivity is less straightforward since they lack a clearly defined topographic property (both channels correspond to their spatial locations). To address the issue of creating cluster‐forming rules for channel combinations for connectivity metrics we used graphs, i.e., in a similar fashion to how channels and their connectivity (spatial and functional) can be modelled as graphs, channel combinations can also be modelled as graphs. Our rationale and procedure are described below.

**FIGURE 1:**
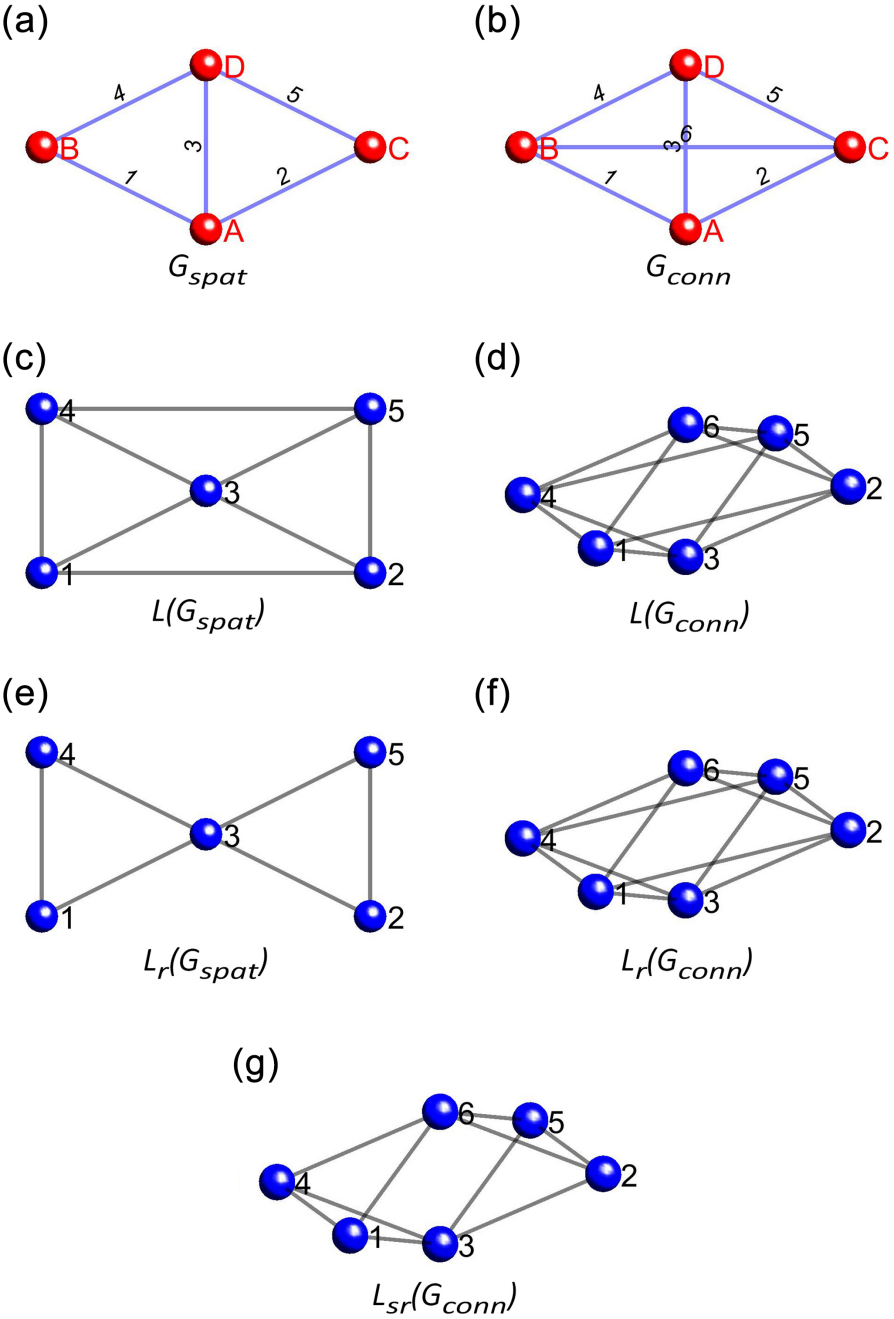
Clustering of connectivity based on graph theory. (a) Hypothetical layout of signal visualised as a $G_{\text{spat}}$. The red nodes marked with upper‐cased letters correspond to the signal locations, while the blue edges marked with numbers correspond to the spatial relationship of the signal locations. (b) Connectivity to be tested for the signals in $G_{\text{spat}}$ visualised as a $G_{\text{conn}}$. The red nodes marked with upper‐cased letters correspond to the same signal locations as in $G_{\text{spat}}$, while the blue edges marked with numbers correspond to the functional connectivity to be tested. (c) and (d) represents the line‐graphs of $G_{\text{spat}}$ and $G_{\text{conn}}$, respectively. The blue nodes correspond to the edges of the original graphs, while the grey lines correspond to the egde‐adjacencies (i.e., neighbourhoodness of edges). (e) and (f) represents the line‐graphs of $G_{\text{spat}}$ and $G_{\text{conn}}$, respectively, with adjacency restricted to edges with adjacent non‐shared nodes. As you can see, edges 4 and 5 of $G_{\text{spat}}$ are not adjacent anymore because their non‐shared nodes (b and c) are also not adjacent. It does not change the line‐graph of $G_{\text{conn}}$ because all connectivity between all signals is of interest, therefore, all nodes are connected to all nodes. (g) represents the final solution, where the edge‐adjacency for $G_{\text{conn}}$ is restricted based on the node‐adjacency in $G_{\text{spat}}$. As a result, edges 4 and 5 of $G_{\text{conn}}$ are not adjacent anymore.

Let us begin with a standard graph where the nodes represent channels, and the edges link any pair of channels either spatially ($G_{\text{spat}}$, Figure 1a) or via hypothesised functional connectivity ($G_{\text{conn}}$, Figure 1b). Adjacencies between the edges of a graph G can be found using the line‐graph representation $L\left(G\right)$, where each node in $L\left(G\right)$ corresponds to an edge in G (Harary & Norman, 1960) ($L\left(G_{\text{spat}}\right)$ and $L\left(G_{\text{conn}}\right)$, Figure 1c,d). More explicitly, if two edges in G are incident on a common node, they will be connected via an edge in $L\left(G\right)$, that is, they will be adjacent. A further restriction on the edge‐adjacency in an$\;L\left(G\right)$ can be applied, whereby two edges can only be adjacent if the non‐shared nodes are also adjacent in G. This additional restriction on edge‐adjacency is based on the rationale that extends the aforementioned observation of data similarity to connections, namely, connections between a channel to a set of channels adjacent to each other are likely to be more similar. The result of this additional restriction is the restricted line graph $L_{r}\left(G\right)$, and we can have one for the spatial connectivity ($L_{r}\left(G_{\text{spat}}\right)$, Figure 1e) and another for the hypothesised functional connectivity ($L_{r}\left(G_{\text{conn}}\right)$, Figure 1f).

This additional restriction of edge‐adjacency can be generalised so that the restricted edge‐adjacency of a hypothesised functional connectivity graph $L_{r}\left(G_{\text{conn}}\right)$ is based on the node‐adjacency in a spatial graph $G_{\text{spat}}$. More explicitly, two edges can only be adjacent in $L_{r}\left(G_{\text{conn}}\right)$ if the non‐shared nodes are also adjacent in $G_{\text{spat}}$. This concept is demonstrated in Figure 1g, wherein the line‐graph of the fully connected hypothesised functional connectivity $L\left(G_{\text{conn}}\right)$ is restricted by the spatial graph $G_{\text{spat}}$ to return a spatially restricted line graph $L_{\mathrm{sr}}\left(G_{\text{conn}}\right)$. Looking closely, one can see that nodes 1 and 2 of $L_{\mathrm{sr}}\left(G_{\text{conn}}\right)$ (which are edges in the original hypothesised functional connectivity graph $G_{\text{conn}}$), are not connected because even though they share node A in $G_{\text{conn}}$, the non‐shared nodes (B and C) are not adjacent in the spatial graph $G_{\text{spat}}$. For inferencing on connectivity, the cluster‐forming rule is defined based on the spatially restricted line graph $L_{\mathrm{sr}}\left(G_{\text{conn}}\right)$.

## 3 RESULTS

### 3.1 Demographics and behavioural data

**FIGURE 2:**
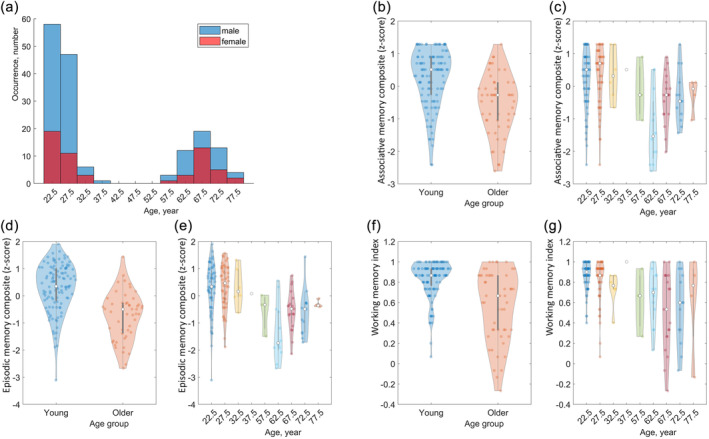
Demographics and memory performance of the final sample. (a) Distribution of age and gender. (b), (d) and (f) show the distribution of performance in associative (b), episodic (d) and working (f) memory for the participants labelled as “young” and “older”. (c), (e) and (g) show the distribution of performance in the same memory domains for the participants in the original age groups. In plots (b–g), the dots represent individual performances.

The final sample included 163 participants, 112 (33 females) in the “young” group and 51 (24 females) from the “older” group. The sample distribution across the nine age groups can be observed in Table S1 and Figure 2a.

The composite memory scores are displayed in Figure 2b–g and Table S2 for both the “young” and “older” groups, and for the nine age groups. Wilcoxon rank sum tests were performed to compare the composite associative (AM), episodic (EM), and working (WM) memory scores between the “young” and the “older” group. The composite memory scores were significantly larger in younger than older adults' groups in every domain, with a very large effect size, AM (Figure 2b): *W* = 4233.5, *p* < .001, *r* = 0.48; EM (Figure 2d): *W* = 4591, *p* < .001, *r* = 0.61; WM (Figure 2f): *W* = 4235.5, *p* < .001, *r* = 0.48. Considering the nine pre‐defined age groups the Kruskal–Wallis rank sum test also resulted in significant differences with small to medium effects, AM (Figure 2c): χ^2^(8) = 28.61, *p* < .001, *r* = 0.18; EM (Figure 2e): χ^2^(8) = 40.99, *p* < .001, *r* = 0.25; WM (Figure 2g): χ^2^(8) = 31.18, *p* < .001, *r* = 0.19.

### 3.2 Age‐related changes in mEOEC power and EO/EC reactivity and relation to memory performance

#### 3.2.1 Age‐related changes in frequency and the effects of band individualisation on power

**FIGURE 3:**
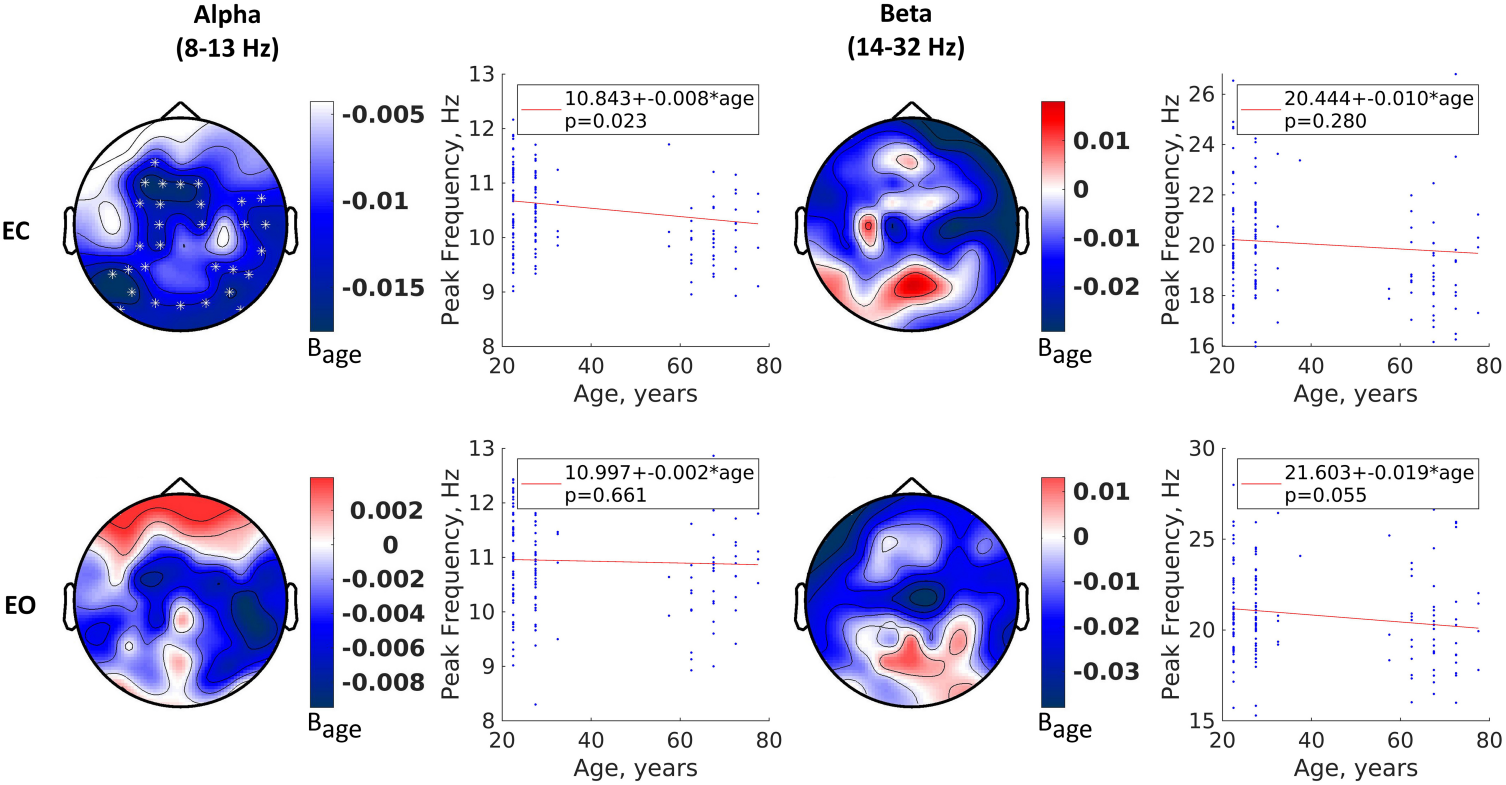
Age‐related changes in alpha and beta peak frequency. The topoplots demonstrate the localised effect of age on the peak frequencies for alpha and beta and for eyes‐closed (EC) and eyes‐open (EO) conditions. The values correspond to the slope (B) of the regression analysis. The white stars correspond to a significant effect. The line and scatter plots demonstrate the effect of age on the same peak frequencies averaged over all channels. The red lines correspond to the regression line, while the blue dots correspond to the individual peak frequencies.

We first investigated whether individual peak frequencies within alpha and beta frequency ranges varied with age. The topographic distribution of the effect of ageing revealed global changes in peak frequency for both alpha and beta frequencies, with somewhat weaker effects in the frontal regions for alpha and the posterior midline regions for both alpha and beta (Figure 3, topoplots). This effect was statistically significant only for alpha in the EC condition in the frontal midline, occipital, and right temporal regions. The analysis of peak frequencies revealed an age‐related downward shift in individual alpha and beta across sensors (Figure 3, regression plots, data were averaged across all sensors for each individualised frequency band). This shift was larger in the EC conditions for frequencies in the alpha range (EC: 0.008 Hz/year, EO: 0.002 Hz/year) and in the EO condition for frequencies in the beta range (EC: 0.01 Hz/year, EO: 0.019 Hz/year), reaching significance for alpha EC (*t*(161) = −2.294, *p* = .023) and near‐significance for beta EO (*t*(161) = −1.935, *p* = 0.055). This indicates that the effects of ageing on peak frequency changes are more robustly detected in the alpha band for the EC condition. This might, in part, be explained by the relatively larger signal‐to‐noise ratio in alpha during EC (Figure S2). Importantly, there is a large between‐subject variance, which is even larger than the effect of ageing, which explains only 2–3% of the variance in the data. These results highlight the importance of condition‐specific individualisation beyond simply scaling with age.

**FIGURE 4:**
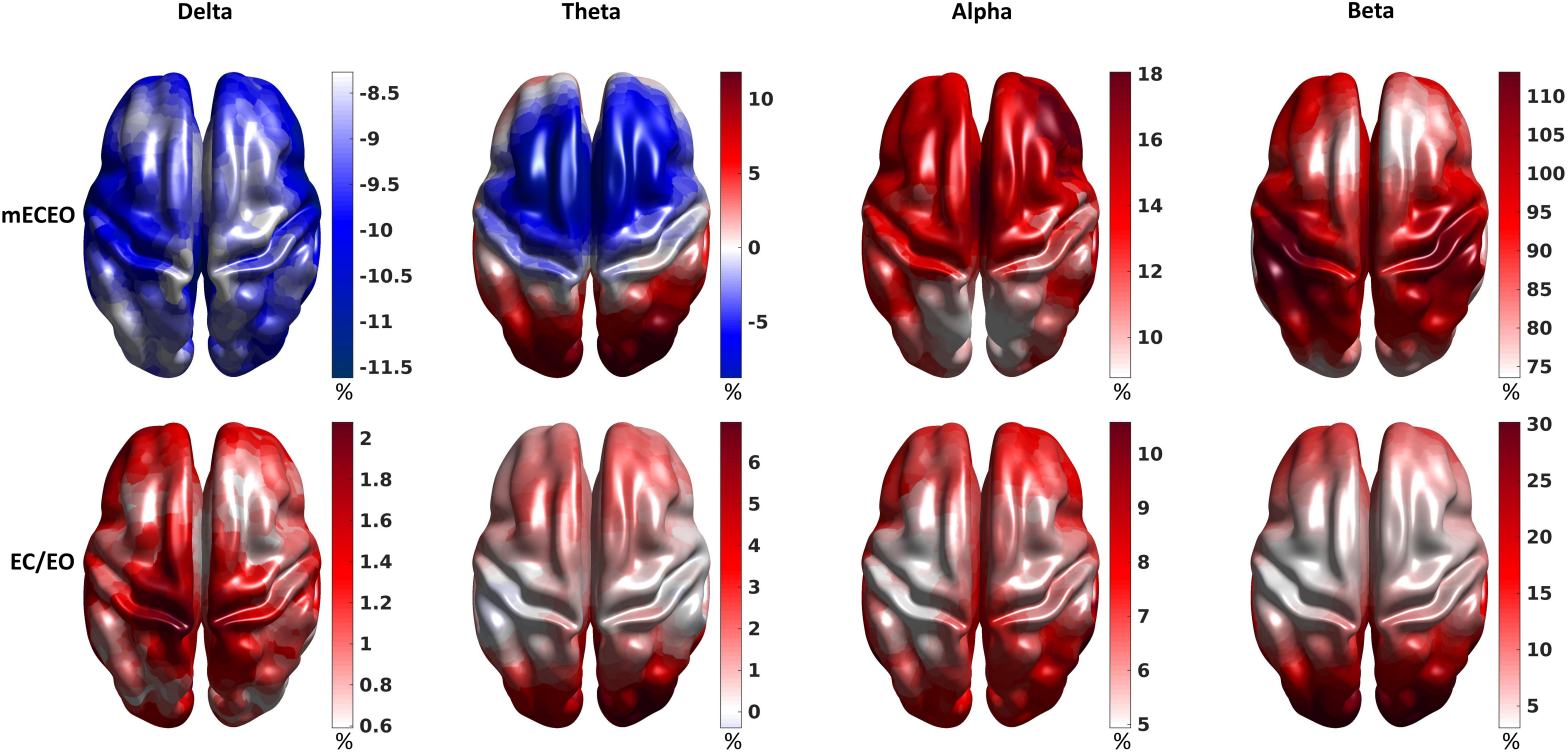
The effect of band individualisation on the combined neural measures. The surface plots demonstrate the localised effect of band individualisation on the combined neural measures of mean power (mECEO) and reactivity (EC/EO). The values correspond to how much the measures change (in % of the measures estimated in the canonical bands) after individualisation.

Next, we investigated the effect of individualised frequency ranges on power estimates by comparing the relative differences observed between the individually determined and canonical bands. Individualised alpha and beta bands were centred at the individual alpha and beta peak frequencies ± the estimated half‐bandwidth, delta and theta bands were shifted to ensure there was no overlap between the bands while keeping their canonical bandwidth. Compared with standard bands, band individualisation resulted in around a 10% global reduction in mEOEC power with more emphasis on the frontal and central areas in the delta and theta power and a 10% localised increase in the temporo‐occipital areas in the theta power (Figure 4, top left half). On the other hand, band individualisation strongly increased the alpha and beta power, resulting in up to 18% and 110% increase, respectively, with a maximum effect in the frontal areas (alpha) and the temporo‐parieto‐occipital junction (beta) (Figure 4, top right half). The band individualisation also affected the reactivity in power, although with a smaller effect. There was an up to 2% global increase in delta reactivity with more emphasis on the central regions, while there was an up to 7% increase in theta reactivity in the occipital regions (Figure 4, bottom left half). The alpha and beta reactivity increases were larger, up to 10% and 30%, respectively, and primarily included the frontal and posterior regions (Figure 4, bottom right half). Overall, these results support the importance of considering individually defined frequency bands when exploring age‐related changes in brain function.

#### 3.2.2 Age‐related changes in mean power and reactivity

**FIGURE 5:**
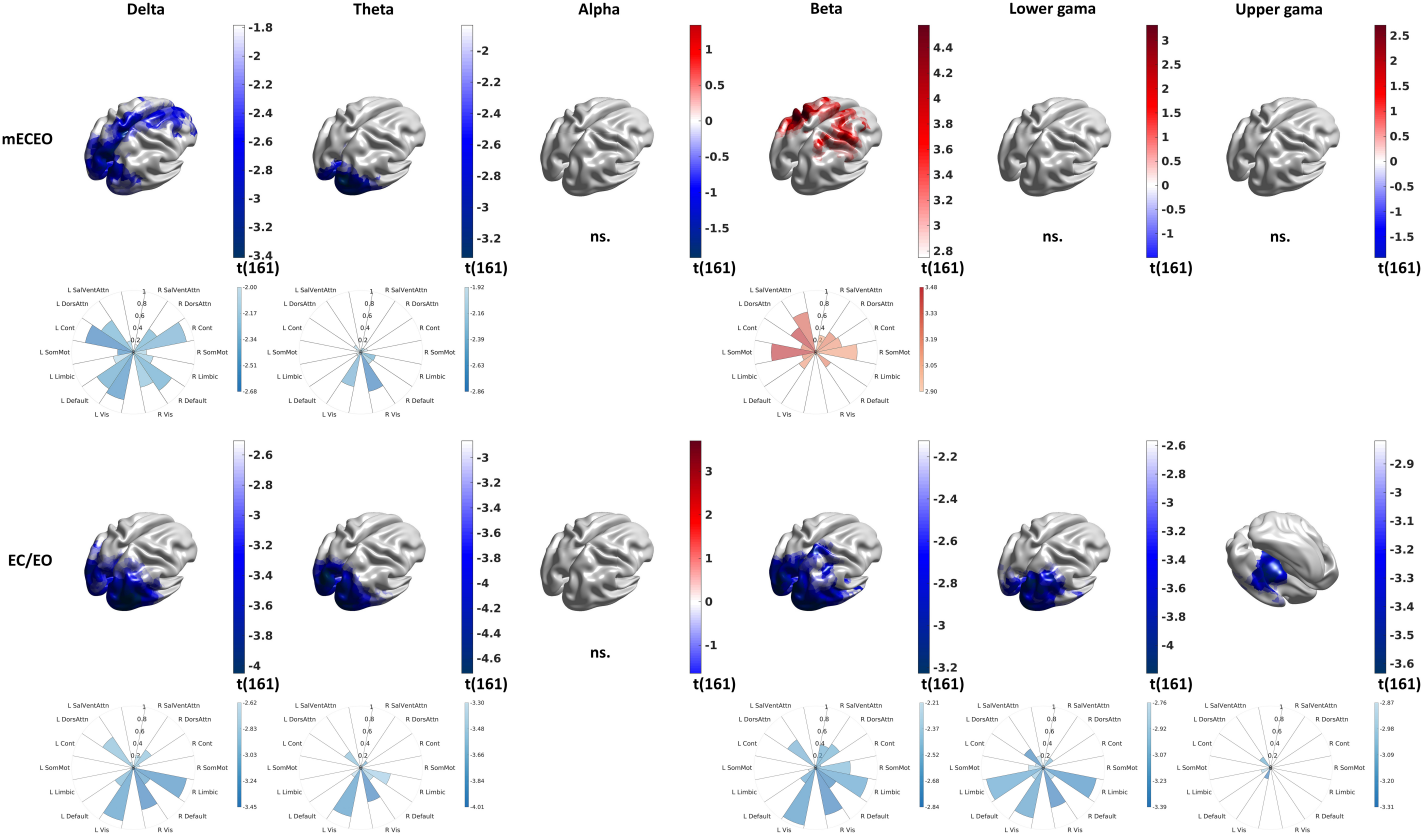
Age‐related changes in the combined neural measures. The surface plots demonstrate the significant localised effect of ageing, measured as t‐statistics of the regression, on the combined neural measures of mean power (mECEO) and reactivity (EC/EO) in the individualised bands. “ns” denotes cases with no significant effect. The polar plots visualise the detected effects averaged in the seven functionally annotated networks in both hemispheres. The colour corresponds to the effect size as measured with the t‐statistics of the regression, while the size of the wedges corresponds to the proportion of the functional network involved.

Our results for mean power and power reactivity are displayed in Figure 5 for individualised bands and in Figure S3 for canonical frequency bands; Table 1 summarises the age‐related changes per frequency band (individualised and canonical) for each brain network.

**TABLE 1:** The effect of age and band individualisation on the combined neural measures.

	SalVentAttn		DorsAttn		Cont		SomMot		Limbic		Default		Vis	
	mECEO	EC/EO	mECEO	EC/EO	mECEO	EC/EO	mECEO	EC/EO	mECEO	EC/EO	mECEO	EC/EO	mECEO	EC/EO
Delta			C + I	C + I	C + I					C + I	C + I		C + I	C + I
Theta													C + I	I
Alpha				C										
Beta	C + I		I	I			C + I	C + I		I				I
Lower Gamma										C + I				C + I
Upper Gamma				C + I						C + I				C + I

*Note*: The table summarises in which functionally annotated networks we detected age‐related changes in mean power (mECEO) and reactivity (EC/EO) by using canonical (C) and individualised (I) bands.

Abbreviations: C, canonical bands; I, individualised bands.

We observed an age‐related reduction in mEOEC delta power in the occipital and midfrontal regions corresponding to the Visual, Default, Control, and Dorsal Attention networks. Delta power reactivity showed more focal changes in the Visual, Dorsal Attention, and Limbic networks. Here, band individualisation made only a minor difference (Figure 5 vs. Figure S3). We found age‐related reductions of mean theta power and reactivity in the occipital regions corresponding to the Visual network and no changes in the midline regions. Here, band individualisation strongly increased the sensitivity to detect the ageing effect in theta reactivity. We saw no effect in alpha power or reactivity. Here, band individualisation made only a minor difference by pushing a very focal effect of ageing below the significance threshold in alpha reactivity in the right prefrontal region. Ageing affected beta mEOEC power and reactivity differently, increasing mEOEC beta power in the Somatomotor and Dorsal and Ventral Attention networks while reducing beta reactivity in the Visual, Limbic, Somatomotor, and Dorsal Attention networks. Band individualisation strongly increased the sensitivity to detect ageing effects in beta power. We saw an age‐related reduction in reactivity in the lower gamma band in the Visual and Limbic networks. Finally, we observed some age‐related reductions in reactivity in the upper gamma band in the Visual, Dorsal Attention, and Limbic networks.

#### 3.2.3 Mean power and reactivity correlates of memory

**FIGURE 6:**
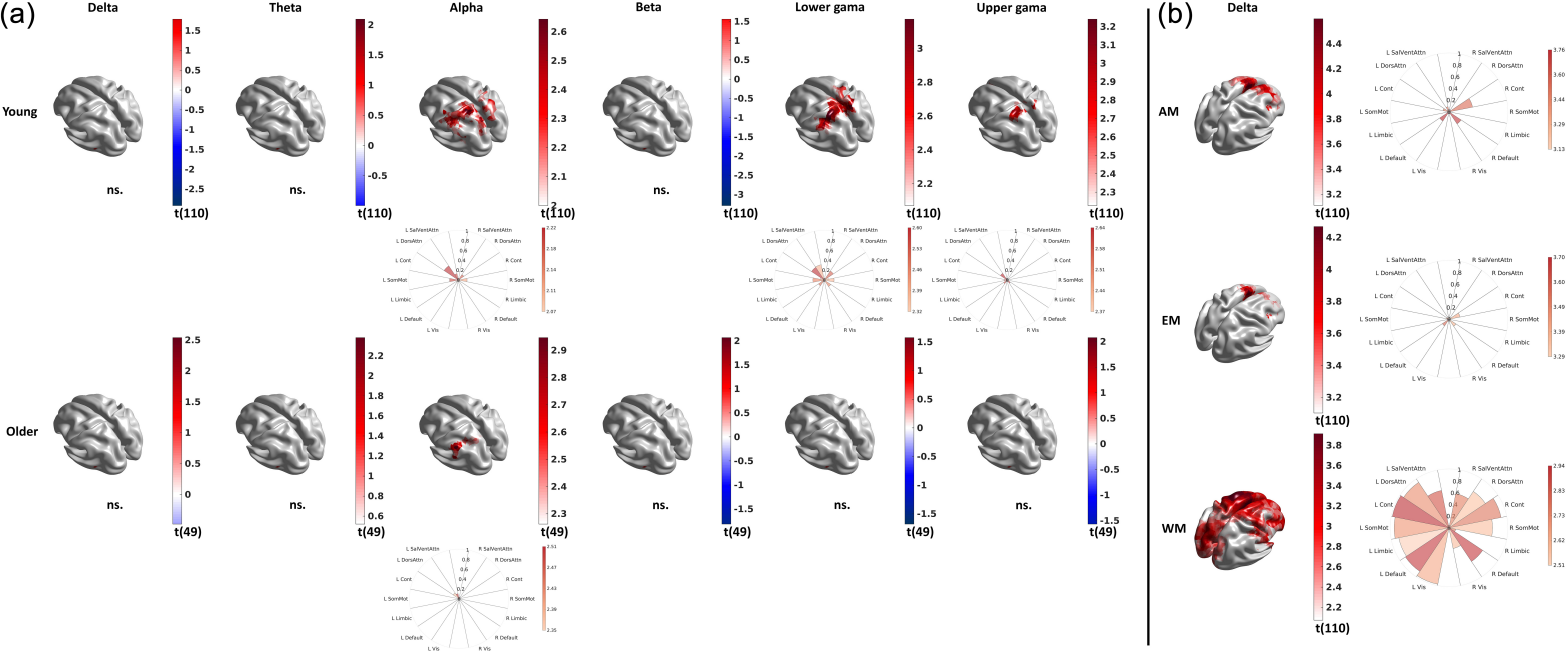
Neural correlates of memory. (a) The surface plots show brain areas where mean power (mECEO) in the individualised bands has a significant relationship with the working memory performance of the “younger” (upper row) and the “older” (lower row) participants. (b) The surface plots show brain areas where reactivity (EC/EO) in the individualised delta band has a significant relationship with associative (AM), episodic (EM), and working memory (WM) performance of the “younger” participants. “ns” denotes cases with no significant effect. The polar plots visualise the detected effects averaged in the seven functionally annotated networks in both hemispheres. The colour corresponds to the effect size as measured with the t‐statistics of the regression, while the size of the wedges corresponds to the proportion of the functional network involved.

Analyses of the relationship between memory performance and the mean power estimates only showed significant correlations with performance in working memory for both young and older adults (Figure 6). mEOEC power in individualised bands was positively correlated with working memory performance in the alpha band for both young and older adults. The topography showed overlapping left‐lateralised correlation in both groups in regions belonging to the Dorsal Attention network, occupying a larger area in the young group (Figure 6a and Table 2). Furthermore, younger adults also showed significantly positive correlations with a similar topography for lower and upper gamma activity. Overall, this supports previous findings on the importance of the Dorsal and Ventral Attention networks and alpha and gamma activity in working memory (Jokisch & Jensen, 2007; Majerus et al., 2018).

**TABLE 2:** The effect band individualisation on the neural correlates of working memory.

	SalVentAttn		DorsAttn		Cont		SomMot		Limbic		Default		Vis	
	Y	O	Y	O	Y	O	Y	O	Y	O	Y	O	Y	O
Delta														
Theta				C						C			C	C
Alpha			I	C + I										
Beta														
Lower Gamma	C + I		C + I											
Upper Gamma			C + I											

*Note*: The table summarises in which functionally annotated networks we detected significant relationship between mean power (mECEO) and working memory performance of the “young” (Y) and the “older” (O) participants by using canonical (C) and individualised (I) bands.

Abbreviations: C, canonical bands; I, individualised bands; O, older; Y, young.

When performing the same analyses for the canonical frequency bands, we observed, in addition, a positive relationship between mEOEC theta power and working memory performance for both young and older adults (Figure S4 and Table 2). Band individualisation eliminated this relationship and improved the sensitivity to detect the abovementioned relationship in the alpha range.

Analyses of the relationship between memory performance and power reactivity estimates showed significantly positive relationships across all memory domains, but only for delta frequency and in the young adults' group (Figure 6b). This relationship was observed in the right Control and the bilateral Default networks in all three memory functions. For working memory, there was a widely distributed correlation with delta reactivity. Band individualisation improved sensitivity in general and led to more significant test statistics and somewhat larger extent of the engagement of the various networks (Figure S4B).

### 3.3 Age‐related changes in connectivity in mEOEC and EO/EC reactivity and relation to memory performance

#### 3.3.1 Band individualisation effects on connectivity

Band individualisation affected connectivity estimates more drastically than power estimates. It eliminated all age‐related and some of the WM‐related variations in cross‐frequency connectivity. For consistency and simplicity, in the rest of the article, we will only discuss cases where age‐ and memory‐related findings were present for canonical and individualised bands.

#### 3.3.2 Age‐related changes in connectivity

**FIGURE 7:**
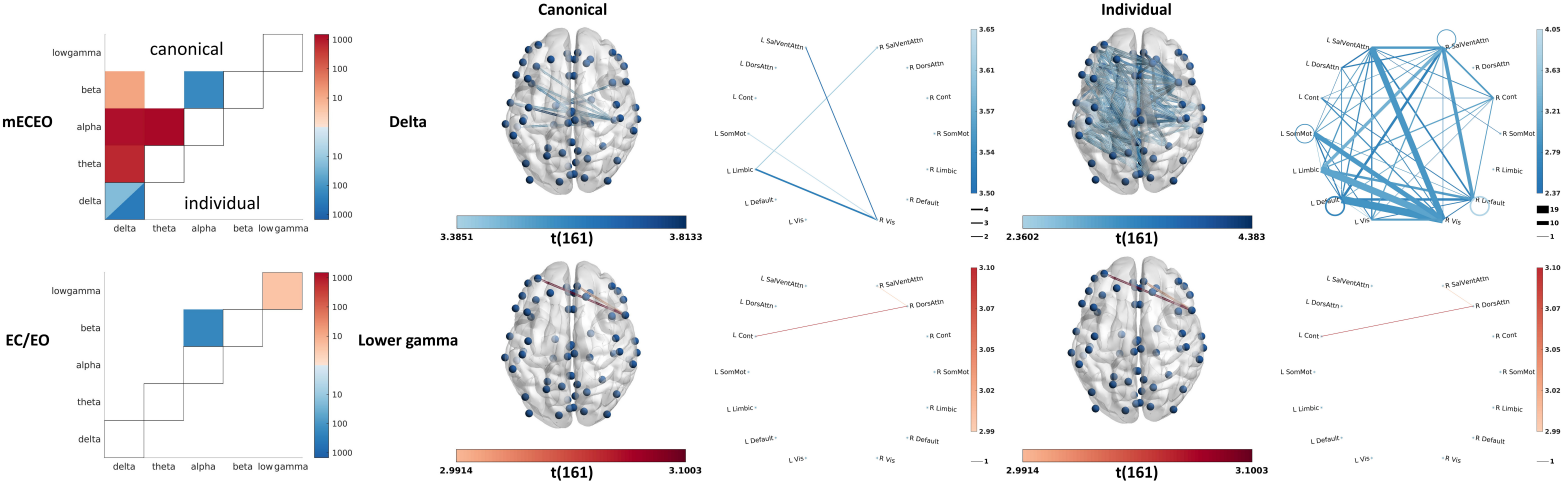
The effect of age and band individualisation on the combined connectivity measures. The matrix plots summarise the number of connections with significant age‐related changes as detected by using canonical (upper triangle) and individualised (lower triangle) bands. Red and blue colours correspond to positive and negative effects, respectively. Within‐frequency connectivity is visualised in the diagonal region, while cross‐frequency connectivity is visualised in the off‐diagonal regions. For the latter, only lower‐to‐higher frequency connectivity was estimated. For mean connectivity (mECEO), only the delta band showed significant age‐related reduction also after band‐individualisation, which is further visualised on the surface and the network plots.

Ageing led to a widespread reduction in mEOEC delta connectivity, with a stronger emphasis on the cross‐hemispheric connections and a focal increase in the reactivity in lower gamma connectivity between the Control and Dorsal and Ventral Attention networks (Figure 7, and Figures S5 and S6 for corresponding connectivity patterns separated by age group). Band individualisation strongly increased the sensitivity (in the delta band), revealing a more distributed reduction in the connectivity, especially between the hemispheres (102 intra vs. 266 cross‐connections). The involved connections form a network with the right Visual network (41% of the connections) as the main hub.

#### 3.3.3 Connectivity correlates of memory

**FIGURE 8:**
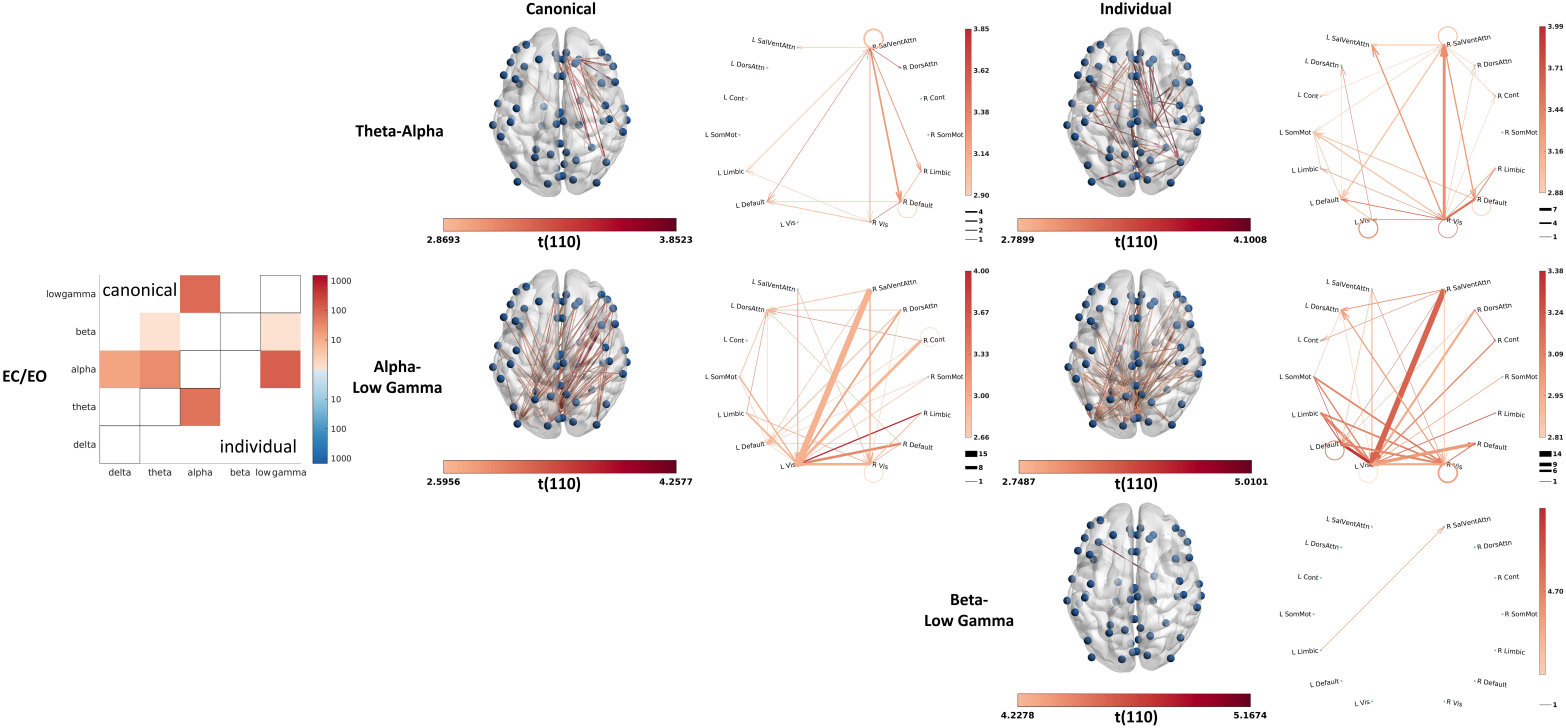
Connectivity correlates of working memory. The matrix plots summarise the number of connections with significant relationship with the working memory performance of the “young” participants as detected by using canonical (upper triangle) and individualised (lower triangle) bands. Red and blue colours correspond to positive and negative effect, respectively. Within‐frequency connectivity (none detected) is visualised in the diagonal region, while cross‐frequency connectivity is visualised in the off‐diagonal regions. For the latter, only lower‐to‐higher frequency connectivity was estimated. Only reactivity (EC/EO) in theta‐alpha, alpha‐lower gamma, and beta‐lower gamma connectivity showed a significant positive relationship with the working memory performance of “young” participants also after band‐individualisation, which are further visualised on the surface and the network plots. The cross‐frequency connectivity is directed (lower to higher frequency), and the direction of the connectivity is marked with arrowheads.

Analyses of the relationship between memory performance and the reactivity of connectivity revealed the involvement of several networks in working memory, however, only in the young group (Figure 8). The reactivity of the theta‐alpha, alpha‐lower gamma, and beta‐lower gamma connectivity across several networks showed to be increased in those with better working memory. The theta‐alpha connections were rather intra‐hemispheric (38 intra vs. 22 cross) with right‐sided dominance (9 left vs. 29 right). The connectivity pattern formed a network with the right Ventral Attention (38% of the connections) and Visual (48% of the connections) networks as the main modulating hubs (62% and 93% of their connections are outputs, respectively). The alpha‐lower gamma connections, however, were rather cross‐hemispheric (42 intra vs. 70 cross) with some within‐hemispheric connections particularly in the left hemisphere (33 left vs. 9 right). The connections for alpha‐lower gamma formed a network with the left Visual (63% of the connections) network as the main receptive hub (80% of its connections are input).

## 4 DISCUSSION

In this study, we used the open‐access LEMON dataset to investigate electrophysiological markers of healthy ageing and memory function. Our study confirmed the impact of individually defined frequency bands on electrophysiological neuromarkers of ageing and cognitive function. Moreover, we also identified several resting state electrophysiological correlates of memory function, particularly when focusing on dynamic, that is, reactivity estimates of power and functional connectivity. Notably, our findings are presented using functionally annotated brain networks to improve interpretability, we deploy graphs to perform cluster correction for functional connectivity and we provide a sharable and reproducible pipeline for electrophysiological data analysis.

### 4.1 Methodological implications

A robust finding in the literature is a slowing in alpha frequency with age (Cesnaite et al., 2023; Klimesch, 1999; Tröndle et al., 2023). The same observation was replicated here for the eyes closed condition where the alpha amplitude is naturally larger, and matching the condition typically investigated in previous ageing studies (Babiloni et al., 2006; Breslau et al., 1989; Polich, 1997; Tröndle et al., 2023; Vysata et al., 2012). This age‐related shift in peak alpha frequency has important methodological implications, specifically that canonical alpha bands are not appropriate for investigating age‐related changes in alpha power (Tröndle et al., 2023) or connectivity (Clark et al., 2004; Jabès et al., 2021; Knyazeva et al., 2018; Lodder & van Putten, 2011; Peltz et al., 2010). Our study confirms these findings and further suggests the importance of considering between‐participant and ‐condition variability when adjusting bands. This band individualisation has a moderate‐to‐strong effect on potential neuromarkers. It moderately reduced delta and theta and strongly increased alpha and beta power (Figure 4). Despite applying the individual band boundaries uniformly across the channels for each participant, we observed some topographic heterogeneity, especially for the alpha and beta bands, with a stronger effect in the posterior areas. More importantly, the effect of band individualisation on the reactivity indicates that EC and EO conditions are affected differently. This difference is negligible in the delta and theta bands (<3%) and moderate in the alpha (8%–18%) and beta bands (5%–40%). The topographic heterogeneity of the effect further supports an interaction between location and condition. These results underpin the importance of condition‐specific band individualisation, which influences the sensitivity of identifying neural correlates of ageing and cognitive performance.

Since individual peaks were reliably detected only for the alpha and beta bands, corresponding differences, that is, age‐related increase in beta power (Figure 5 vs. Figure S3) and neural correlates of working memory in the alpha power (Figure 6 vs. Figure S4), are not surprising. However, the downward shift of the alpha band can also affect the neighbouring theta band, which explains the increase in sensitivity of detecting age‐related reduction in theta reactivity (Figure 5 vs. Figure S3). The effect of band individualisation on connectivity is even more drastic. It eliminated most of the findings while enabling greater sensitivity in detecting an age‐related increase in within‐delta connectivity and some of the neural correlates of working memory in the reactivity of cross‐frequency connectivity (Figures 7 and 8).

The visual comparison of the magnitude of the effect of band individualisation on power and connectivity suggests that power measures are more robust and forgiving of methodological choices that do not account for individual variations in peak frequencies, as observed by the smaller differences between the two sets of findings. Connectivity, however, was more sensitive to methodological choice; therefore, the construction of any workflow analysing connectivity requires more careful consideration of potential confounds. This also implies that findings on connectivity‐based neuromarkers are more likely to change with the advancement of the field. Because methodological flexibility can lead to inconsistent findings and lack of reproducibility (Lejko et al., 2020; Mahjoory et al., 2017) we used a reproducible and shareable pipeline (Automatic Analysis) to build a transparent workflow.

### 4.2 Confirmatory findings on age‐related electrophysiological changes

The pace of reduction in alpha frequency with age (0.5 Hz from 20‐ to 80‐year‐old) was between what has been previously reported 0.45 Hz (Tröndle et al., 2023) to 1 Hz (Donoghue et al., 2020; Knyazeva et al., 2018). This slowing was observed throughout the scalp, especially in the midfrontal and occipital regions, with a larger effect on the right hemisphere, similar to Tröndle et al. (2023). Slowing in alpha frequency might reflect changes in the level of neurotransmission and excitation‐inhibition ratios, as well as a decrease in axonal conduction velocity (Dustman et al., 1993; Hong & Rebec, 2012). A more controversial finding in ageing research pertains to changes in alpha power, with previous findings indicating either a decline with age (Babiloni et al., 2006; Barry & de Blasio, 2017; Kumral et al., 2020), or no age‐related alterations (Sahoo et al., 2020) particularly when controlling for the reduction in alpha peak frequency or non‐oscillatory 1/*f* slope of the EEG (Caplan et al., 2015; Cesnaite et al., 2023). Our findings agree with the later evidence of no changes in alpha power when defining individualised bands centred on individual alpha frequency.

We observed a reduction in delta and theta and an increase in beta power (mEOEC), similar to the first study analysing this dataset with a slightly different methodology (Kumral et al., 2020). The topography of age‐related changes in mean power was somewhat similar to Kumral et al., 2020. However, there were also some notable differences, potentially due to a different estimation of power and our use of data acquired in both the EO and EC conditions. In contrast with Kumral et al., we found age‐related reductions of mean theta power and reactivity in the occipital regions corresponding to the visual network and no changes in the midline regions. Age‐related changes in theta power are, however, controversial. Previous studies have shown decreases (Vlahou et al., 2014), increases (Babiloni et al., 2006; Ishii et al., 2018; Klass & Brenner, 1995) or no changes (Hong & Rebec, 2012) with age. The observed decrease in delta and theta power with age in our study might, however, be explained by the now well‐replicated flattening of the 1/f slope with age, with a higher impact on lower frequencies (Dave et al., 2018; Lodder & van Putten, 2011; Voytek et al., 2015). Perhaps less controversial is our observation of age‐related increases in beta power, which agrees with previous literature showing higher resting‐state beta activity in older adults (Gómez et al., 2013; Heinrichs‐Graham et al., 2018; Heinrichs‐Graham & Wilson, 2016; Hübner et al., 2018; Koyama et al., 1997; Veldhuizen et al., 1993). The topography of our findings matches previous studies showing higher spontaneous beta power in frontal and parietal regions that form part of the Somatomotor and Salience networks. In particular, the observation of increased power in the Somatomotor network matches previous whole‐brain analysis indicating motor regions as those with higher differences in beta power between young and older cohorts (Heinrichs‐Graham & Wilson, 2016). Changes in beta power at rest are thought to index age‐related changes in GABAergic inhibition (Inamoto et al., 2023; Rossiter et al., 2014).

In regard to age‐related changes in reactivity, we observed a decrease in reactivity across all frequency bands with the exception of the alpha band. In accordance with Barry and de Blasio (Barry & de Blasio, 2017), we also detected an anterior–posterior gradient in the spatial distribution of age‐related changes in the reactivity across several bands. We complement these findings by showing a reduction in gamma reactivity, which was not previously investigated in this dataset but is consistent with similar findings in Jabes et al. (Jabès et al., 2021). Moreover, our findings allow us to observe finer spatial patterns at the functional network level (see below) that were not captured by previous studies with lower spatial resolution.

The analysis of the relationship between connectivity and age revealed several age‐related changes. While most age‐related changes in mEOEC power were not significant when using individualised bands, we observed a robust age‐related decrease in connectivity in the delta band across hemispheres, with most changes affecting the connections of the right visual network. Comparison of resting state EEG of Alzheimer's disease patients and healthy controls led to similar findings suggesting a decline in interhemispheric communication or compensation mechanisms (Hata et al., 2016).

### 4.3 New findings on age‐related electrophysiological changes and memory function

With our more comprehensive analysis, we were also able to complement existing findings and contribute to the debate on the relationship between EEG oscillations and cognitive ageing. We propose that our assessment of the effect of the condition‐specific individualisation adds additional weight to our findings. In particular, we found increased sensitivity in detecting age‐related changes in the beta power, the theta reactivity and the delta connectivity and the neural correlates of working memory in the alpha power and the theta‐alpha and the alpha‐lower gamma connectivity.

Several studies, however, found a link between alpha power and cognitive performance or decline (Babiloni et al., 2022; Fonseca et al., 2011; van der Hiele et al., 2008), which is also consistent with our finding of a positive correlation between alpha (and gamma) power and working memory performance. These indicate that alpha power can be considered a reliable and specific neuromarker of working memory performance irrespective of age.

However, our investigation of the relationship between functional connectivity and memory performance only showed consistent findings in the younger group. In particular, we found that better working memory performance correlated with rest reactivity of theta‐alpha connectivity within and between frontal and parietal regions. This pattern closely resembles changes elicited by working memory tasks (Akiyama et al., 2017). Moreover, the reactivity of both theta‐alpha and alpha‐gamma connectivity has been reported to correlate with attention, processing speed, and executive functions (Siebenhühner et al., 2020), key components of working memory. Our findings also resolve some of the inconsistency around the role of delta oscillations in cognition (Trammell et al., 2017) by showing that it reflects domain‐independent dynamics (i.e., reactivity) rather than static components of memory. The lack of significant correlates of connectivity and memory performance in older adults might be partly explained by the lower sample size in comparison with the effect size in this age group; however, the literature on the relationship between rs‐EEG connectivity and memory is scarce. Hata and coworkers, for example, were able to demonstrate the reduced connectivity within the delta band as a potential neuromarker of dementia rather than healthy memory function in a sample including 28 “probable AD patients” and 30 healthy controls (Hata et al., 2016).

#### 4.3.1 Functionally annotated electrophysiology

Our electrophysiological findings confirmed that ageing affects the activity (power) and reactivity of multiple networks involved in perception and cognition, such as the Control, Dorsal Attention, Visual, and Default networks (Figure 5). The functional annotation draws a richer picture of and provides contextual support and mechanistic understanding for the neural correlates of memory performance, indicating the primary roles of Dorsal and Ventral Attention networks in working memory (Figure 6a) and the primary role of Control networks in associative and episodic memory (Figure 6b). The left hemispheric lateralisation of the neural correlates of working memory (Figure 6a) emphasizes the verbal components (Nagel et al., 2013; Ray et al., 2008) of the working memory task used during the assessment. Our findings on the role of cross‐frequency connectivity in working memory further confirm the sensitivity of neuromarkers related to dynamic (i.e., reactivity) rather than static neural features and revealed some fundamental differences in the engagement of the lower‐ (theta‐alpha) and higher‐ (alpha‐gamma) frequency networks in working memory. The lower‐frequency network predominantly engages the right hemisphere via shorter paths and is driven by the Visual and Ventral Attention network (Figure 8, first row), suggesting integration of information implemented via perception‐driven (i.e., bottom‐up) and salience and subsequent attention‐driven (i.e., top‐down) processes (Akiyama et al., 2017). The higher‐frequency network, on the other hand, is characterised by cross‐hemispheric communication via longer paths modulated by the Visual network (Figure 8, second row) suggesting the dominance of top‐down processes.

Investigating electrophysiological neuromarkers at the source level allows one to identify their correspondence and functional annotation with fMRI findings even without synchronous recording. In addition to improving the interpretability of the EEG findings, this approach also allows to put them into a wider multimodal context.

### 4.4 Limitations

While the LEMON dataset constitutes a powerful resource to study electrophysiological markers of healthy ageing, it currently has some inherent limitations. The first is the absence of participants' ages, as the dataset contains instead nine pre‐defined age groups. Such an arrangement reduces the sensitivity to detect age‐related changes because it does not allow the full modelling of age‐related variability. The second is the overrepresentation of younger participants (≤35 year‐old) in the sample. This was primarily due to the exclusion criteria of the original LEMON study, including medical conditions typically more frequent in the older population. This selection bias was further aggravated by the rejection of samples with artifacts, which was more common in the older group. As a result, our analyses have more power to detect effects in the “young” than in the “older” group; therefore, the lack of an effect in the “older” group cannot be unambiguously interpreted. Thirdly, we omitted the investigation of the effect of sex due to the unbalanced representation of females and males across the age groups and the whole sample. Finally, information about the time of the day in which acquisitions took place was not available and circadian and ultradian rhythms can impact the spectral characteristics of EEG signals (Lehnertz et al., 2021). These limitations might be addressed by the addition of further information and extensions to the LEMON dataset or by combining it with other openly available datasets. The analyses of these datasets will benefit from reproducible pipelines, such as the one we produced for this study.

An additional limitation of our study is that power estimates included both periodic and aperiodic components. While we did account for individual differences when defining frequency bands, a recent study emphasized the importance of decomposing periodic and aperiodic components when investigating the age effect on alpha power during the EC conditions, albeit mentioning that the contribution of the two components to cognitive decline is still unclear (Tröndle et al., 2023). Future studies could investigate how accounting for both individualised bands and aperiodic components contributes to changes in functional connectivity patterns within and between frequency bands and their relation to cognitive performance.

Finally, the statistical framework, as implemented in Fieldtrip, does not support multiple regression. To partially overcome this limitation, we split the participants into “young” and “older” groups, however, it does not allow a full separation of the age‐effect from the correlates of memory functions, somewhat reducing the sensitivity to detect them. The LIMO toolbox allows modelling multiple variabilities in M/EEG data by using hierarchical linear modelling (Pernet et al., 2011). Future developments in our pipeline or Fieldtrip might address this issue and allow the full integration of LIMO.

## 5 CONCLUSION

Despite the recent developments in neuroimaging, which allow sophisticated analyses on larger datasets to extract psychobiologically relevant features to be conducted, we still have limited success in identifying reliable neuromarkers of age‐related cognitive decline. The present article synthesizes best practices and implements methodological considerations to provide an integrative insight into the electrophysiological correlates of age‐related cognitive decline using functionally annotated brain networks. Our analyses confirmed the most established neuromarkers of cognitive ageing, while it also clarified the role of delta and alpha oscillations in memory performance and their changes in ageing. We also highlighted the importance of considering the variation in band specification across individuals and conditions and demonstrated the higher sensitivity of dynamic rather than static electrophysiological neuromarkers in ageing and memory.

## AUTHOR CONTRIBUTIONS

Tibor Auer: conceptualisation, data curation, methodology, software, formal analysis, validation, writing—original draft, writing—review & editing, visualization, supervision; Robin Goldthorpe: conceptualisation, data curation, writing—original draft; Robert Peach: conceptualization, writing—review & editing; Henry Hebron: methodology, software, formal analysis; Ines Violante: conceptualisation, supervision, funding acquisition, project administration, writing—original draft, writing—review & editing.

## FUNDING INFORMATION

This study was supported by the Biotechnology and Biological Sciences Research Council, London (BB/S008314).

## CONFLICT OF INTEREST STATEMENT

None.

## Supporting information


**FIGURE S1.** Data processing and analysis workflow as implemented in Automatic Analysis (aa). The figure represents the data processing and analysis steps and the data flow between them. The steps, implemented as aa modules (aamod_*), are visualised as text in a box, while the data, managed in aa streams, are visualised as text on arrows indicating the direction of the dataflow. The preprocessing steps are common for the canonical and individualised bands. Analysis steps using canonical bands are marked with green, while analysis steps using individualised bands are marked with turquoise.

**FIGURE S2.** Age‐related changes in the power spectral density. The figure shows the power spectral density averaged across all channels in the EC and EO conditions for young and older adults.

**FIGURE S3.** Age‐related changes in the combined neural measures. The surface plots demonstrate the significant localised effect of ageing, measured as t‐statistics of the regression, on the combined neural measures of mean power (mECEO) and reactivity (EC/EO) in the canonical bands. “ns.” denotes cases with no significant effect. The polar plots visualise the detected effects averaged in the seven functionally annotated networks in both hemispheres. The colour corresponds to the effect size as measured with the t‐statistics of the regression, while the size of the wedges corresponds to the proportion of the functional network involved.

**FIGURE S4.** Neural correlates of memory. (a) The surface plots show brain areas where mean power (mECEO) in the canonical bands has a significant relationship with the working memory performance of the “younger” (upper row) and the “older” (lower row) participants. (b) The surface plots show brain areas where reactivity (EC/EO) in the canonical delta band has a significant relationship with associative (AM), episodic (EM), and working memory (WM) performance of the “younger” participants. “ns.” denotes cases with no significant effect. The polar plots visualise the detected effects averaged in the seven functionally annotated networks in both hemispheres. The colour corresponds to the effect size as measured with the t‐statistics of the regression, while the size of the wedges corresponds to the proportion of the functional network involved.

**FIGURE S5.** Mean connectivity in the young and older participants. Mean connectivity in the delta band, estimated as a within‐group one‐sample t‐test of wPLI averaged across the EC and EO conditions, for the young and older adults. The glass brain plots show significant (*p* = .05, Bonferroni‐corrected) connectivity between channel combinations. The circular plots summarise connectivity between the functionally annotated networks. Only positive values (i.e., wPLI >0) were statistically significant.

**FIGURE S6.** Mean reactivity in within‐frequency connectivity for the young and older participants. Reactivity in the within‐frequency mean connectivity in the low gamma band, estimated as a within‐group one‐sample *t*‐test of EC/EO ratio of wPLI, for the young (a) and older (b) adults. The glass brain plots show significant (*p* = .05, uncorrected) connectivity between channel combinations. The circular plots summarise connectivity between the functionally annotated networks. ‘Positive’ and ‘Negative’ denote the relative sign of the t‐values, indicating positive or negative wPLI.

**TABLE S1.** Participant demographics. The table summarises the number of participants divided into sexes and the nine age groups in the final sample after data cleaning.

**TABLE S2.** Memory performance. The table summarises the associative (AM), episodic (EM), and working memory (WM) performance of the “young” and the “older” participants.

## Data Availability

The data that support the findings of this study are openly available in Max Planck Institut Leipzig Mind‐Brain‐Body Dataset‐LEMON at http://fcon_1000.projects.nitrc.org/indi/retro/MPI_LEMON.html.
