# Supplementary figures and images for: Functionally annotated electrophysiological neuromarkers of healthy ageing and memory function
Source: Hum Brain Mapp. 2024 Apr 23;45(6):e26687. doi: 10.1002/hbm.26687 (PMC11036379; doi:10.1002/hbm.26687)

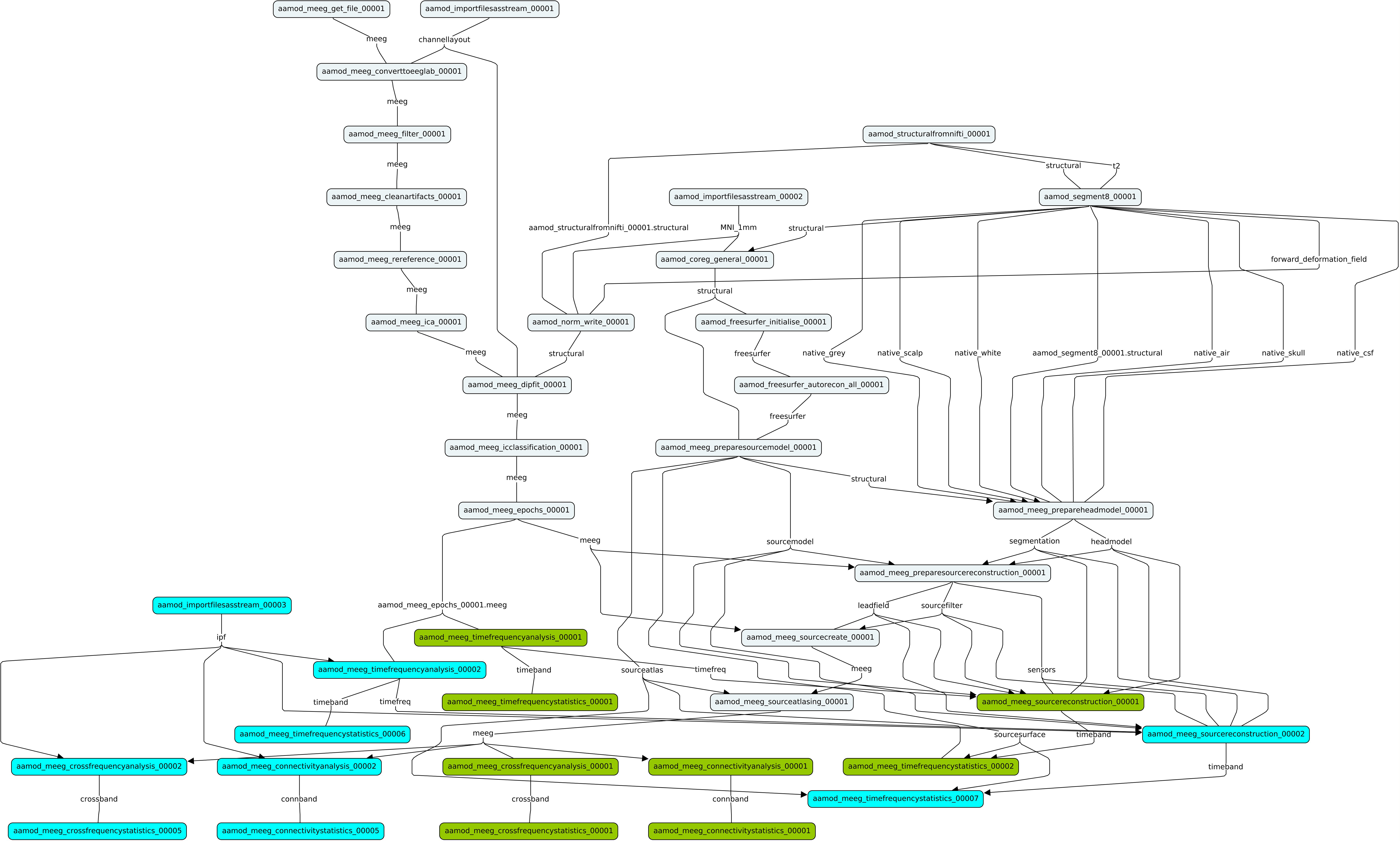

Supplement: Supplementary file 1 — FIGURE S1. Data processing and analysis workflow as implemented in Automatic Analysis (aa). The figure represents the data processing and analysis steps and the data flow between them. The steps, implemented as aa modules (aamod_*), are visualised as text in a box, while the data, managed in aa streams, are visualised as text on arrows indicating the direction of the dataflow. The preprocessing steps are common for the canonical and individualised bands. Analysis steps using canonical bands are marked with green, while analysis steps using individualised bands are marked with turquoise. [file HBM-45-e26687-s002.jpg]

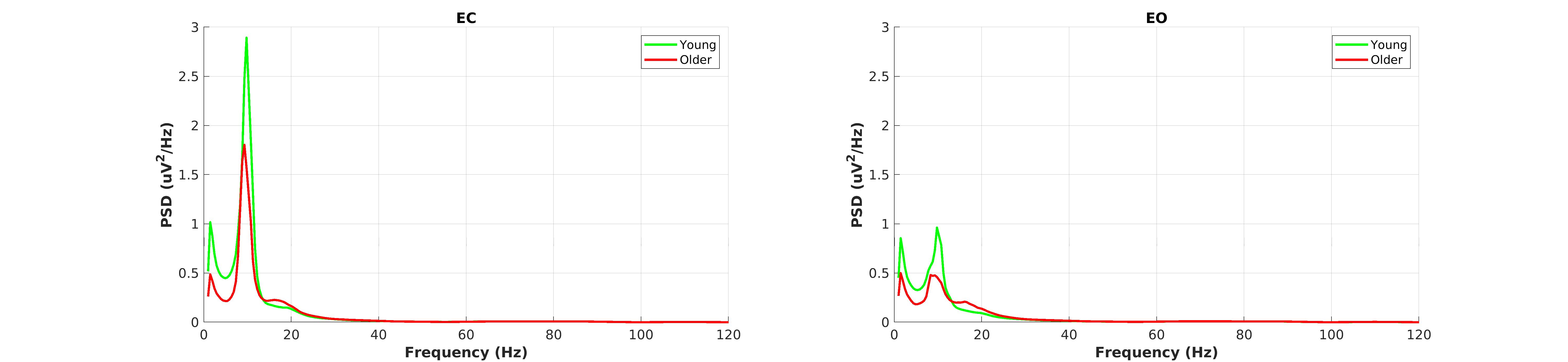

Supplement: Supplementary file 2 — FIGURE S2. Age‐related changes in the power spectral density. The figure shows the power spectral density averaged across all channels in the EC and EO conditions for young and older adults. [file HBM-45-e26687-s004.jpg]

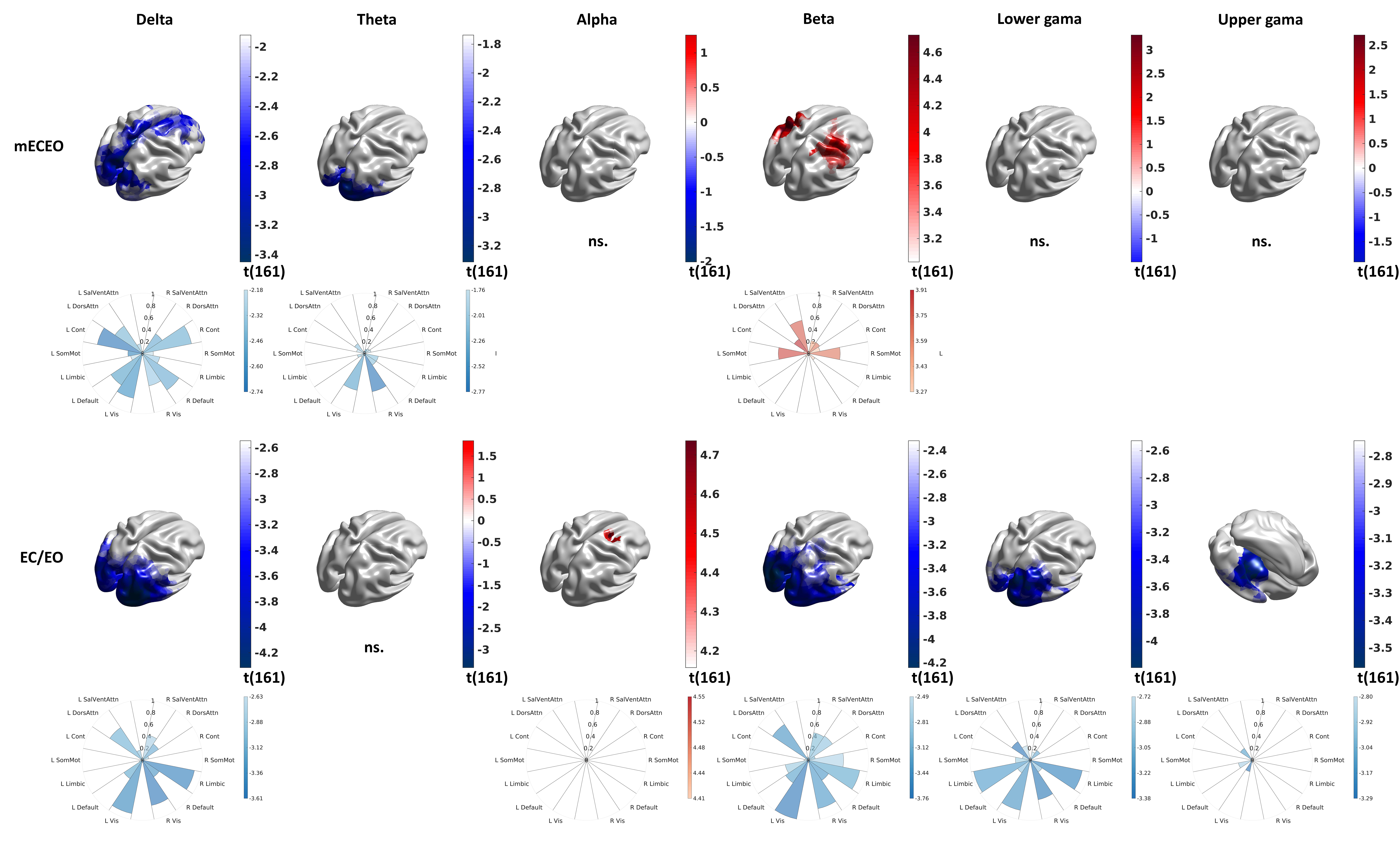

Supplement: Supplementary file 3 — FIGURE S3. Age‐related changes in the combined neural measures. The surface plots demonstrate the significant localised effect of ageing, measured as t‐statistics of the regression, on the combined neural measures of mean power (mECEO) and reactivity (EC/EO) in the canonical bands. “ns.” denotes cases with no significant effect. The polar plots visualise the detected effects averaged in the seven functionally annotated networks in both hemispheres. The colour corresponds to the effect size as measured with the t‐statistics of the regression, while the size of the wedges corresponds to the proportion of the functional network involved. [file HBM-45-e26687-s006.jpg]

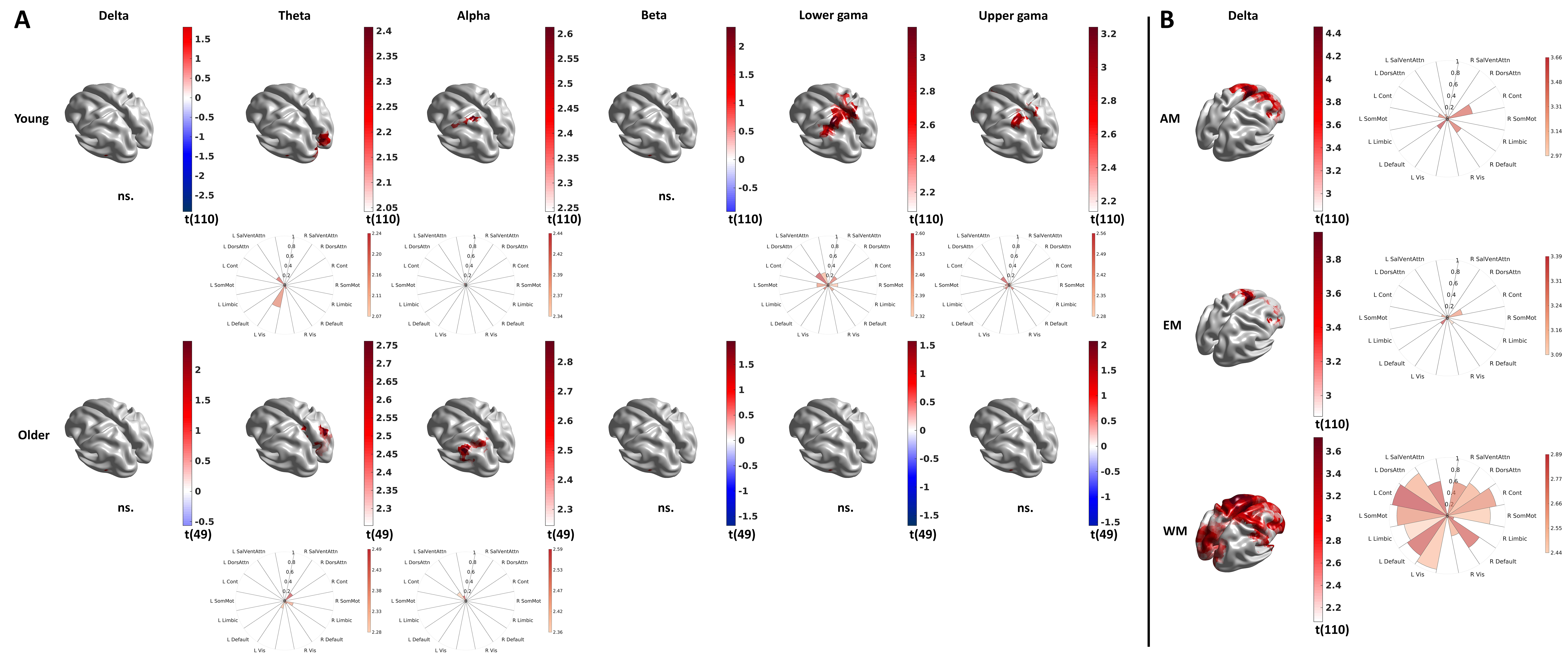

Supplement: Supplementary file 4 — FIGURE S4. Neural correlates of memory. (a) The surface plots show brain areas where mean power (mECEO) in the canonical bands has a significant relationship with the working memory performance of the “younger” (upper row) and the “older” (lower row) participants. (b) The surface plots show brain areas where reactivity (EC/EO) in the canonical delta band has a significant relationship with associative (AM), episodic (EM), and working memory (WM) performance of the “younger” participants. “ns.” denotes cases with no significant effect. The polar plots visualise the detected effects averaged in the seven functionally annotated networks in both hemispheres. The colour corresponds to the effect size as measured with the t‐statistics of the regression, while the size of the wedges corresponds to the proportion of the functional network involved. [file HBM-45-e26687-s005.jpg]

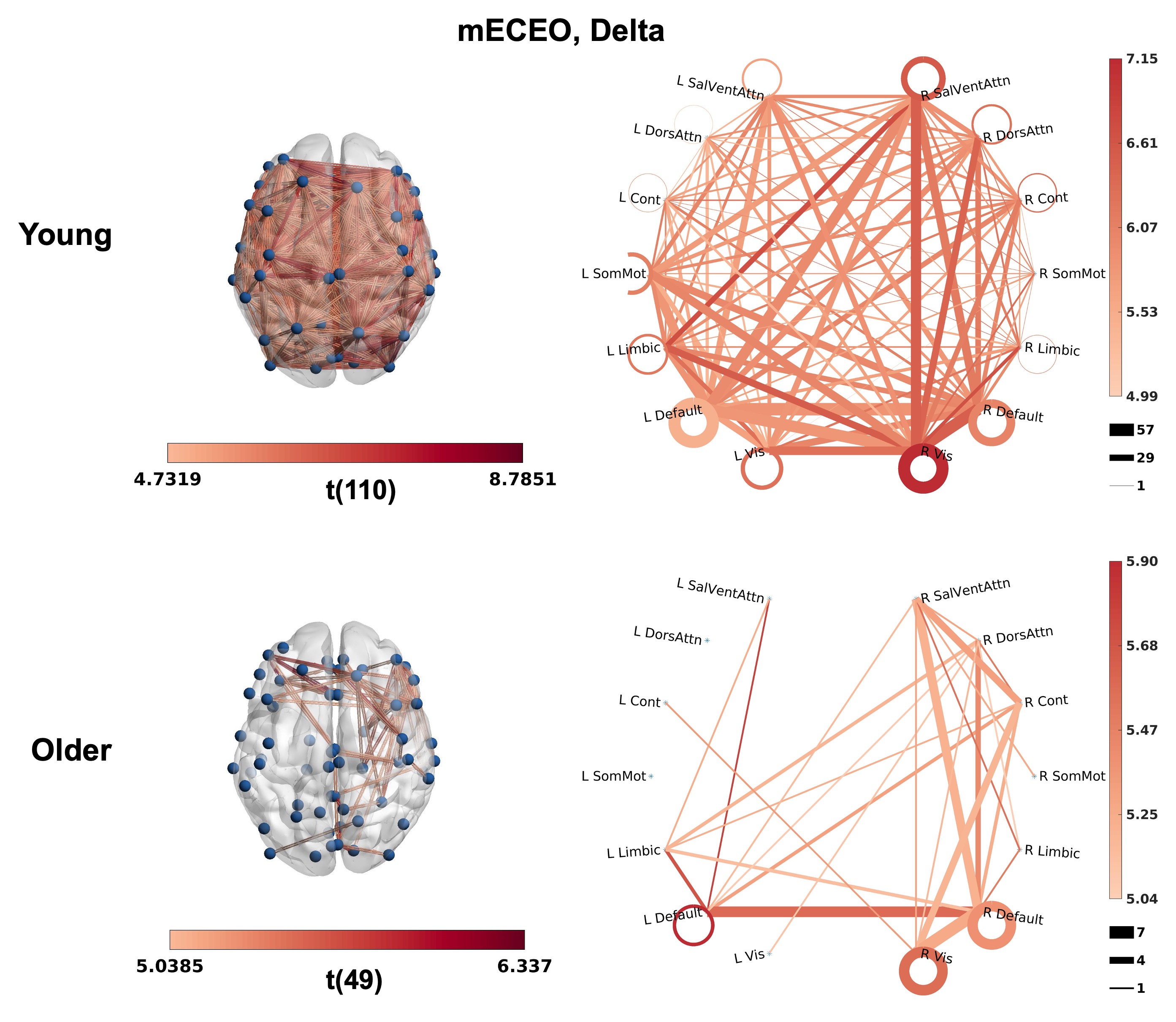

Supplement: Supplementary file 5 — FIGURE S5. Mean connectivity in the young and older participants. Mean connectivity in the delta band, estimated as a within‐group one‐sample t‐test of wPLI averaged across the EC and EO conditions, for the young and older adults. The glass brain plots show significant (p = .05, Bonferroni‐corrected) connectivity between channel combinations. The circular plots summarise connectivity between the functionally annotated networks. Only positive values (i.e., wPLI >0) were statistically significant. [file HBM-45-e26687-s001.jpg]

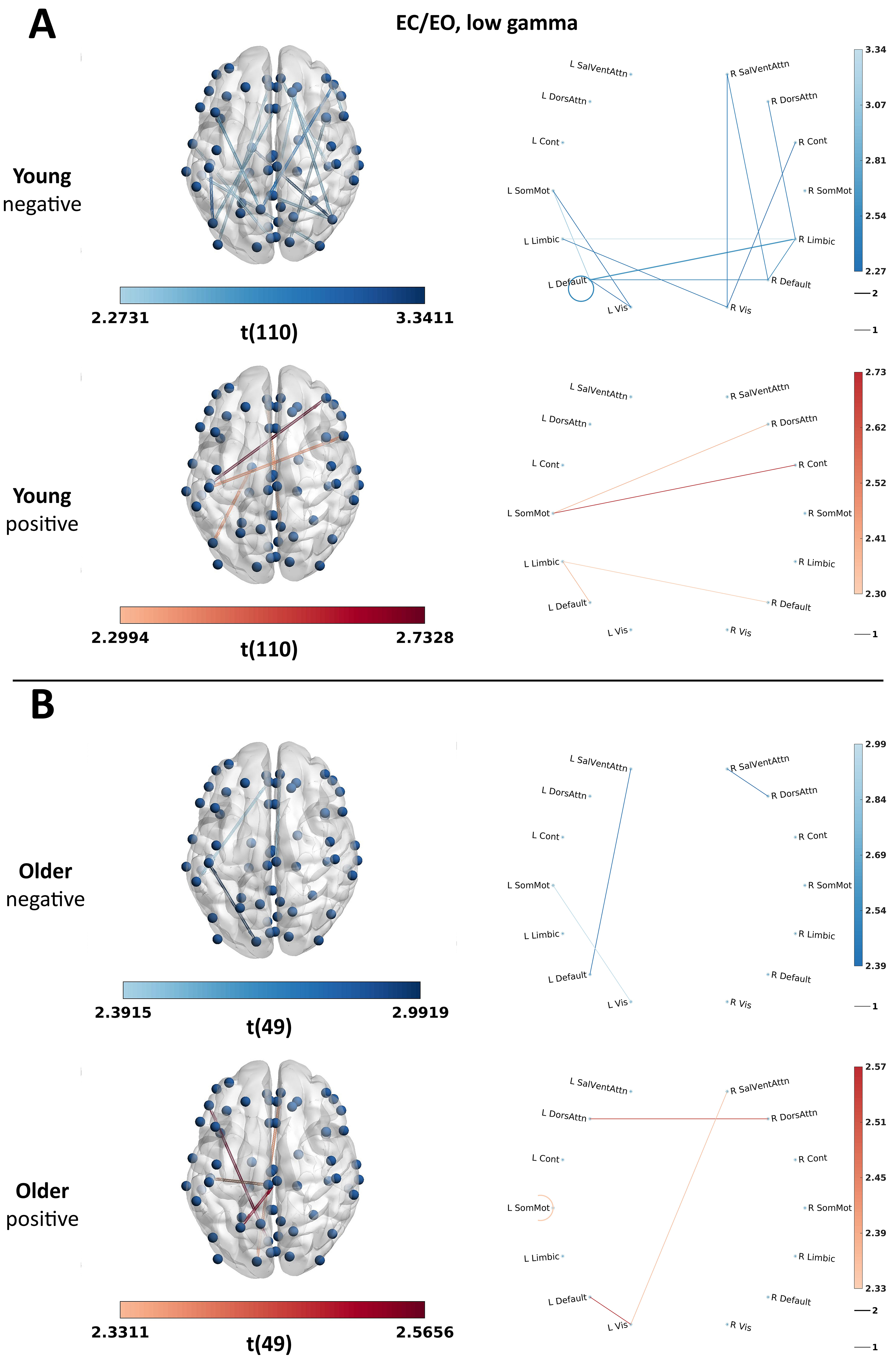

Supplement: Supplementary file 6 — FIGURE S6. Mean reactivity in within‐frequency connectivity for the young and older participants. Reactivity in the within‐frequency mean connectivity in the low gamma band, estimated as a within‐group one‐sample t‐test of EC/EO ratio of wPLI, for the young (a) and older (b) adults. The glass brain plots show significant (p = .05, uncorrected) connectivity between channel combinations. The circular plots summarise connectivity between the functionally annotated networks. ‘Positive’ and ‘Negative’ denote the relative sign of the t‐values, indicating positive or negative wPLI. [file HBM-45-e26687-s007.jpg]
